# Design, Synthesis, and In Vitro Antiproliferative Activity of Hydantoin and Purine Derivatives with the 4-Acetylphenylpiperazinylalkyl Moiety

**DOI:** 10.3390/ma14154156

**Published:** 2021-07-26

**Authors:** Agnieszka Zagórska, Anna Czopek, Anna Jaromin, Magdalena Mielczarek-Puta, Marta Struga, Dorota Stary, Marek Bajda

**Affiliations:** 1 Department of Medicinal Chemistry, Faculty of Pharmacy, Jagiellonian University Medical College, Medyczna 9, 30-688 Kraków, Poland; anna.czopek@uj.edu.pl; 2 Department of Lipids and Liposomes, Faculty of Biotechnology, University of Wroclaw, Joliot-Curie 14a, 50-383 Wroclaw, Poland; 3 Chair and Department of Biochemistry, Medical University of Warsaw, Banacha 1, 02-097 Warsaw, Poland; magdalena.mielczarek-puta@wum.edu.pl (M.M.-P.); mstruga@wum.edu.pl (M.S.); 4 Department of Physicochemical Drug Analysis, Faculty of Pharmacy, Jagiellonian University Medical College, Medyczna 9, 30-688 Kraków, Poland; 1dorota.stary@student.uj.edu.pl (D.S.); marek.bajda@uj.edu.pl (M.B.)

**Keywords:** anticancer activity, thymidine phosphorylase inhibitors, angiogenesis

## Abstract

Cancer represents one of the most serious health problems and the second leading cause of death around the world. Heterocycles, due to their prevalence in nature as well as their structural and chemical diversity, play an immensely important role in anti-cancer drug discovery. In this paper, a series of hydantoin and purine derivatives containing a 4-acetylphenylpiperazinylalkyl moiety were designed, synthesized, and biologically evaluated for their anticancer activity on selected cancer cell lines (PC3, SW480, SW620). Compound **4**, a derivative of 3′,4′-dihydro-2′H-spiro[imidazolidine-4,1′-naphthalene]-2,5-dione, was the most effective against SW480, SW620, and PC3 cancer cell lines. Moreover, **4** has high tumor-targeting selectivity. Based on docking studies, it was concluded that *R* isomers of 3′,4′-dihydro-2′H-spiro[imidazolidine-4,1′-naphthalene]-2,5-dione could be further studied as promising scaffolds for the development of thymidine phosphorylase inhibitors.

## 1. Introduction

Cancer is a disease in which the control of growth is lost in one or more cells, leading to hematological malignancies or a solid mass of cells known as tumors [1]. The World Health Organization (WHO) provides recent information on frequency, mortality, and survival expectancy of the 15 leading types of cancers worldwide. The epidemiological information and the gradual pattern of malignant growth recurrence, commonness, and mortality expected over the next 40 years highlight that cancer will stay a scourge for a long time to come [2]. Moreover, the WHO estimated that, in the next four decades, cancer deaths are expected to overtake those for ischemic heart disease, the current leading cause of global deaths, with a 2.08-fold increase by the year 2060 [3]. The hallmarks of cancer include self-sufficiency in growth signals, insensitivity to growth-inhibitory signals, evasion of apoptosis, limitless replication potential, sustained angiogenesis, tissue invasion and metastasis, and inactivation of systems that regulate cellular response to DNA damage. Treatment options include surgery, radiation therapy, chemotherapy, or immune therapy.

**Figure 1:**
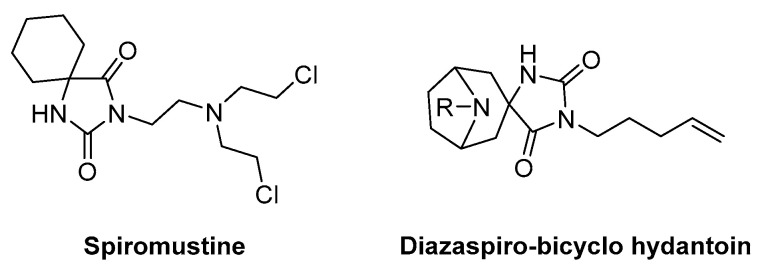
General structures of spiromustine [5] and diazaspiro-bicyclo hydantoin from [8].

Due to their prevalence in nature, as well as their structural and chemical diversity, hydantoins play an immensely important role in anti-cancer drug discovery. While derivatives of hydantoin are commonly used for clinical treatment of epilepsy and cardiac arrhythmias and were recently identified also as antiplasmodial agents [4], hydantoins have also been associated with antitumor activities [5,6,7]. By way of example, spiromustine (Figure 1), a spirohydantoin mustard, penetrates the blood–brain barrier and localizes in brain tumors [5]. Additionally, diazaspiro-bicyclo hydantoins with either an alkene, ester or ether substituents at the N-3 position exhibited antiproliferative effects on K562 and CEM human leukemia cell lines [8]. Moreover, spirohydantoin derivatives containing different side chains at the N-3 (acetic acid propyl ester, methoxy ethane, pentene) and N-8 (phenyl ring with electronegative atoms) positions have been shown to inhibit the growth of leukemic cells [9].

**Figure 2:**
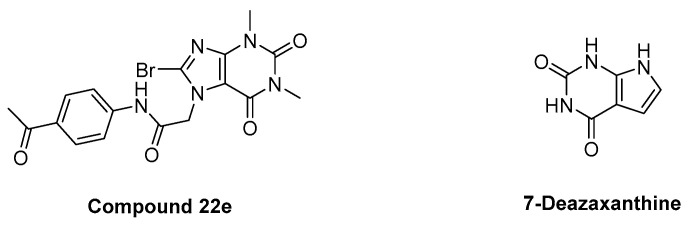
Structures of compound **22e** from [14] and 7-deazaxanthine.

**Figure 3:**
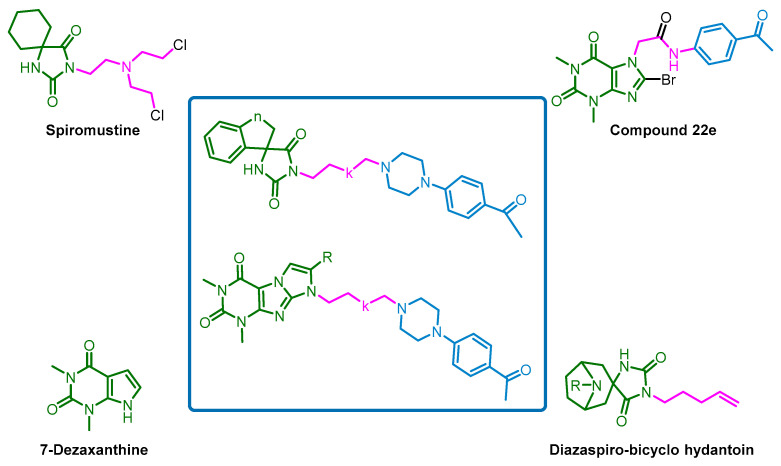
Structures of the designed compounds **1**–**14** based on active anticancer compounds (green—an amide fragment with hydantoin or purine core; blue—an arylamine fragment; magenta—an alkyl linker).

Purine is an important pharmacophore interacting with the synthesis and function of nucleic acids and enzymes. Purine analogs are used, among others, in the treatment of acute leukemias (thiopurines, pentostatin) [10,11], as immunosuppressive agents (azathioprine) [12], or as antitumor agents (olomoucine) [13]. An evaluation of the anticancer activity of purine derivatives via screening against a panel of 60 human cancer cell lines revealed that N-(4-acetylphenyl)-2-[8-bromo-1,3-dimethyl-2,6-dioxo-2,3-dihydro-1H-purin-7(6H)-yl]acetamide (compound **22e**, Figure 2), at a dose of 10 µM, exhibited moderate growth inhibition [14]. Angiogenesis is a vital step in the process of cancer growth; thus, its inhibition could inhibit cancer growth [15]. Thymidine phosphorylase is a pro-angiogenic factor that catalyzes the reversible phosphorolysis of thymidine into thymine and 2′-deoxy-D-ribose 1-phosphate [16]. 7-Deazaxanthine (7-DX, Figure 3), was identified amongst the purine derivatives as the first potent inhibitor of thymidine phosphorylase (TP) and angiogenesis. 7-DX creates extra stabilizing interactions with TP and efficiently inhibits neovascularization [17].

In this paper, two series of nitrogen-containing heterocyclic compounds, based on hydantoin and purine scaffolds were designed, synthesized, and biologically evaluated for their anticancer action on selected cancer cell lines (PC3, SW480, SW620). Series I was designed as a series of spiromustine analogs, where indane and tetralin moieties were implemented in place of the cyclohexane ring. In Series II, the imidazole ring was annulated on a purine-2,4-dione moiety, to obtain the rigid analogue of tipiracil, a member of the pyrimidone class of thymidine phosphorylase inhibitors (Figure 3). A common element to both series of compounds was the 4-acetylphenylpiperazinylalkyl moiety. Compounds based on arylpiperazine have been reported to inhibit the growth of three different prostatic tumor cell lines, namely, PC-3, DU-145, and LNCaP [18]. To avoid potential interactions with G Protein-Coupled Receptors, which is characteristic of long-chain arylpiperazine derivatives, we decided to introduce an acetyl group in the 4-position of the phenyl ring. Moreover, such a moiety is present in compound **22e** [14], which exhibits moderate growth inhibition of a of melanoma cancer cells. For the most active compound from series I and II, namely, compound **4**, the hemolytic activity, TP inhibition and docking studies to the active TP site were determined.

## 2. Materials and Methods

### 2.1. Chemistry

All the reagents were purchased from commercial suppliers: Sigma-Aldrich (Saint Louis, MO, USA), Merck (Darmstadt, Germany), Chempur (Piekary Śląskie, Poland), Fluorochem (Hadfield, UK), Acros Organics (Hampton, VA, USA), Apollo Scientific (Bredbury, UK) and were used without further purification. Analytical thin-layer chromatography (TLC) was performed on Merck Kieselgel 60 F_254_ (0.25 mm) precoated aluminum sheets (Merck, Darmstadt, Germany) using the following mixtures of solvents: (S_1_) methylene chloride/methanol (9:0.7), (S_2_) petroleum ether/ethyl acetate (5:5), (S_3_) methylene chloride/methanol (9:1), and (S_4_) methylene chloride/methanol (9:1.2). Compounds were visualized with UV light and a 2.9% solution of ninhydrin in acetone. Column chromatography was performed using silica gel (particle size 0.063–0.200 mm; 70–230 Mesh ATM) purchased from Merck (Darmstadt, Germany). The microwave reactions were conducted in a DiscoverLabMate (CEM Corporation, Matthews, NC, USA). The UPLC-MS or UPLC-MS/MS analyses were done on an UPLC-MS/MS system comprising a Waters ACQUITY UPLC (Waters Corporation, Milford, CT, USA) coupled with a Waters TQD mass spectrometer (electrospray ionization mode ESI with tandem quadrupole). UPLC separations were carried out according to the procedures described elsewhere [19,20,21,22]. The UPLC/MS purity of all the test compounds and key intermediates was determined to be >95%. NMR spectra for compounds **1**–**14** were obtained in a Varian Mercury spectrometer (Varian Inc., Palo Alto, USA) operating at 300 MHz (^1^H NMR) or 75 MHz (^13^C NMR) and, for compounds **VIII**, **1**–**4**, in a FT-NMR 500 MHz spectrometer (Jeol Ltd., Tokyo, Japan), using CDCl_3_ or DMSO-*d*_6_ as solvent. Chemical shifts are reported as δ values (ppm) relative to TMS δ = 0 (^1^H) as the internal standard. The *J* values are expressed in hertz (Hz). Signal multiplicities are represented by the following abbreviations: s (singlet), br. s (broad singlet), d (doublet), dd (doublet of doublets) dt (doublet of triplets) t (triplet), q (quartet), and m (multiplet). Elemental analyses were conducted using a Vario EL III elemental analyzer (Elementar Analysen Systeme GmbH, Langenselbold, Germany). Melting points were determined on a Büchi Melting Point B-540 (Büchi Labortechnik, Essen, Germany) apparatus using open glass capillaries and are uncorrected.

#### 2.1.1. General Procedure for Obtaining Spirohydantoin (**I**–**IV**)

General procedure for obtaining starting spirohydantoin compounds: 2′,3′-dihydrospiro[imidazolidine-4,1′-indene]-2,5-dione (**I**), 3′,4′-dihydro-2′H-spiro[imidazolidine-4,1′-naphthalene]-2,5-dione (**II**), 1′,3′-dihydrospiro[imidazolidine-4,2′-indene]-2,5-dione (**III**), 3′,4′-dihydro-1′H,2H,5H-spiro[imidazolidine-4,2′-naftalene]-2,5-dion (**IV**) as well as detailed analytical data were described previously [19,20].

#### 2.1.2. General Procedure for Alkylation of Spirohydantoin (**V**–**XII**)

The analytical data of intermediate products 1-(4-bromobutyl)-2′,3′-dihydrospiro[imidazolidine-4,1′-indene]-2,5-dione (**V**), 1-(4-bromobutyl)-3′,4′-dihydro-2′H-spiro[imidazolidine-4,1′-naphthalene]-2,5-dione (**VI**), (1-(4-bromobutyl)-3′,4′-dihydro-1′H-spiro[imidazolidine-4,2′-naphthalene]-2,5-dione (**VII**), 1-(5-bromopentyl)-2′,3′-dihydrospiro[imidazolidine-4,1′-indene]-2,5-dione (**IX**), and 1-(5-bromopentyl)-3′,4′-dihydro-2′H-spiro[imidazolidine-4,1′-naphthalene]-2,5-dione (**X**) were previously described [19,20]. Other intermediate products (***VIII***, **XI**, **XII**) were obtained as the aforementioned structures, but with slight modifications. Briefly: a mixture of the appropriate spirohydantoin (**I**–**IV**; 2.5 mmol) and potassium carbonate (0.69 g, 5 mmol) in acetonitrile (15 mL) was heated to 80 °C on a magnetic stirrer. After 30 min heating, an alkylating agent: 1,4-dibromobutane (0.5938 g, 2.7 mmole,) or 1.5-dibromopentane 0.632 g, 2.7 mmole) was added dropwise to the reaction mixture, which was kept at 80 °C for 20 h. After filtration of the reaction mixture, the filtrate was concentrated under vacuum. The intermediates were purified by column chromatography on silica gel, using (S_1_) and (S_2_) as eluents.

#### 2.1.3. (R,S)-1-(4-Bromobutyl)-1′,3′-dihydro-2H,5H-spiro[imidazolidine-4,2′-indene]-2,5-dione (**VIII**)

Creamy powder. Yield: *64%*; TLC: *R_f_* = *0.69 (S_2_)*; HPLC: *t_R_* = *1.541*; LC/MS: *C_15_H_17_BrN_2_O_2_* (96%) *m/z*: *337.21*, found: *337.07*; ^1^H NMR (500 MHz, CDCL_3_-*d*) δ ppm 1.79–1.92 (m, 4 H, CH_2_C*H*_2_C*H*_2_CH_2_) 3.05 (d, *J* = 16.61 Hz, 2 H, indane) 3.42–3.46 (m, 2 H, (CH_2_)_3_C*H*_2_) 3.58 (t, *J* = 6.59 Hz, 2 H, C*H*_2_(CH_2_)) 3.62 (d, *J* = 16.04 Hz, 2 H, indane) 5.70 (s, 1 H, N_1_*H*_hyd_) 7.19–7.24 (m, 4 H, Ar).

#### 2.1.4. (R,S)-1-(5-Bromopentyl)-1′,3′-dihydro-2H,5H-spiro[imidazolidine-4,2′-indene]-2,5-dione (**XI**)

Creamy powder. Yield: *46%*; TLC: *R_f_* = *0.72 (S_2_)*; HPLC: *t_R_* = *1.536*; LC/MS: *C_16_H_19_BrN_2_O_2_* (*95*%) *m/z*: *351.24*, found: *351.26*; ^1^H NMR (300 MHz, CDCL_3_-*d*) δ ppm 1.41–1.53 (m, 2 H, (CH_2_)_2_C*H*_2_(CH_2_)_2_) 1.63–1.74 (m, 2 H, (CH_2_)_3_C*H*_2_CH_2_) 1.84–1.96 (m, 2 H, CH_2_C*H*_2_(CH_2_)_3_) 3.05 (d, *J* = 16.16 Hz, 2 H, indane) 3.41 (t, *J* = 6.67 Hz, 2 H, (CH_2_)_4_C*H*_2_) 3.55 (t, *J* = 7.18 Hz, 2 H, C*H*_2_(CH_2_)_4_) 3.62 (d, *J* = 16.41 Hz, 2 H, indane) 5.79 (s, 1 H, N_1_*H*_hyd_) 7.18–7.25 (m, 4 H, Ar).

#### 2.1.5. (R,S)-1-(5-Bromopentyl)-3′,4′-dihydro-1′H,2H,5H-spiro[imidazolidine-4,2′-naftalene]-2,5-dione (**XII**)

Creamy powder. Yield: *68%*; TLC: *R_f_* = *0.67 (S_1_)*; HPLC: *t_R_* = *1.641*; LC/MS: *C_17_H_21_BrN_2_O_2_* (*96*%) *m/z*: *365.26*, found: *365.12*; ^1^H NMR (300 MHz, CDCL_3_-*d*) δ ppm ^1^H NMR (300 MHz, CDCL_3_-*d*) δ ppm 1.39–1.53 (m, 2 H, (CH_2_)_2_C*H*_2_(CH_2_)_2_) 1.62–1.73 (m, 2 H, (CH_2_)_3_C*H*_2_CH_2_) 1.82–1.95 (m, 3 H, CH_2_C*H*_2_(CH_2_)_3_, tetraline) 2.16–2.29 (m, 1 H, tetraline) 2.73 (dd, *J* = 16.54, 2.18 Hz, 1 H, tetraline) 2.83–2.97 (m, 1 H, tetraline) 2.99–3.11 (m, 1 H, tetraline) 3.32–3.45 (m, 3 H, (CH_2_)_4_C*H*_2_, tetraline) 3.53 (t, *J* = 7.18 Hz, 2 H, C*H*_2_(CH_2_)_4_) 5.65 (s, 1 H, N_1_*H*_hyd_) 7.04–7.09 (m, 1 H, Ar) 7.11–7.20 (m, 3 H, Ar).

### 2.2. General Procedure for Obtaining the Final Compounds ***1***–***8***

The appropriate bromoalkyl derivative of spirohydantoin (0.3 mmol), potassium carbonate (0.083 g, 0.6 mmol), and a catalytic amount of potassium iodide were dissolved in 10 mL of acetone and 4′-(1-piperazinyl)acetophenone (0.067 g, 0.33 mmol) was added. The reaction mixture was heated to 60 °C and the course of the reaction was monitored by TLC. After 20 h of heating, the reaction mixture was filtered, and concentrated under vacuum. The resultant final compounds were further purified by column chromatography using appropriate eluent systems.

#### 2.2.1. (R,S)-1-(4-(4-(4-Acetylphenyl)piperazin-1-yl)butyl)-2′,3′-dihydrospiro[imidazolidine-4,1′-indene]-2,5-dione (**1**)

Creamy powder. Yield: *71*%; mp 180.2–181.3 °C; TLC: *R_f_* = *0.67 (S_3_)*; HPLC: *t*_R_ = *0.955*; LC/MS: *C_27_H_32_N_4_O_3_* (100%) *m/z*: 460.57, found: *461.38*; ^1^H NMR (500 MHz, CDCl_3_-*d*) δ (ppm): 1.52–1.59 (m, 2 H, CH_2_C*H*_2_(CH_2_)_2_) 1.66–1.74 (m, 2 H, (CH_2_)_2_C*H*_2_CH_2_) 2.19–2.28 (m, 1 H, indane) 2.39–2.46 (m, 2 H, (CH_2_)_3_C*H*_2_) 2.49 (d, *J* = 2.29 Hz, 3 H, C*H*_3_) 2.51–2.61 (m, 4 H, N_1pip_(C*H*_2_)_2_) 2.66–2.73 (m, 1 H, indane) 2.99–3.08 (m, 1 H, indane) 3.24 (dt, *J* = 16.04, 8.02 Hz, 1 H, indane) 3.33 (d, *J* = 4.01 Hz, 4 H, N_4pip_(C*H*_2_)_2_) 3.56 (td, *J* = 7.02, 3.15 Hz, 2 H, C*H*_2_(CH_2_)_3_) 6.01 (br. s., 1 H, N_1_*H*_hyd_) 6.80–6.89 (m, 2 H, Ar) 7.10 (d, *J* = 7.45 Hz, 1 H, Ar) 7.19–7.24 (m, 1 H, Ar) 7.28–7.33 (m, 2 H, Ar) 7.84 (dd, *J* = 8.88, 2.00 Hz, 2 H, Ar). ^13^C NMR (126 MHz, CDCl_3_-*d*) δ (ppm): 23.87, 26.15, 26.20, 30.28, 37.11, 38.53, 47.30, 52.85, 57.89, 71.32, 113.41, 122.66, 125.61, 127.43, 127.58, 129.68, 130.46, 140.14, 144.23, 154.23, 157.02, 175.68, 196.71.

#### 2.2.2. (R,S)-1-(5-(4-(4-Acetylphenyl)piperazin-1-yl)pentyl)-2′,3′-dihydrospiro[imidazolidine-4,1′-indene]-2,5-dione (**2**)

Creamy powder. Yield: *55*%; mp 155.3–156.2 °C; TLC: *R_f_* = *0.67 (S_3_)*; HPLC: *t*_R_ = *0.993*; LC/MS: *C_28_H_34_N_4_O_3_* (96%) *m/z*: 474.59, found: *475.34*; ^1^H NMR (500 MHz, CDCl_3_-*d*) δ (ppm): 1.37 (quin, *J* = 7.73 Hz, 2 H,m CH_2_)_2_C*H*_2_(CH_2_)_2_) 1.58–1.65 (m, 2 H, (CH_2_)_3_C*H*_2_CH_2_) 1.70 (dt, *J* = 14.89, 7.45 Hz, 2 H, CH_2_C*H*_2_(CH_2_)_3_) 2.19–2.28 (m, 1 H, indane) 2.42 (br. s., 2 H, (CH_2_)_4_C*H*_2_) 2.51 (s, 3 H, C*H*_3_) 2.53–2.68 (m, 4 H, N_1pip_(C*H*_2_)_2_) 2.68–2.74 (m, 1 H, indane) 3.05 (ddd, *J* = 16.04, 9.16, 3.44 Hz, 1 H, indane) 3.25 (dt, *J* = 16.04, 8.02 Hz, 1 H, indane) 3.38 (br. s., 4 H, N_4pip_(C*H*_2_)_2_) 3.55 (t, *J* = 7.16 Hz, 2 H, C*H*_2_(CH_2_)_4_) 5.59 (s, 1 H, N_1_*H*_hyd_) 6.85 (d, *J* = 9.16 Hz, 2 H, Ar) 7.10 (d, *J* = 8.02 Hz, 1 H, Ar) 7.21–7.24 (m, 1 H, Ar) 7.29–7.32 (m, 2 H, Ar) 7.86 (d, *J* = 9.17 Hz, 2 H, Ar). ^13^C NMR (126 MHz, CDCl_3_-*d*) δ (ppm): 24.60, 26.19, 26.22, 28.04, 30.28, 37.07, 38.54, 47.23, 52.87, 58.39, 71.30, 113.40, 113.75, 122.65, 125.57, 127.41, 127.54, 129.64, 130.46, 130.89, 140.20, 144.23, 154.22, 157.09, 175.74, 196.75.

#### 2.2.3. (R,S)-1-(4-(4-(4-Acetylphenyl)piperazin-1-yl)butyl)-3′,4′-dihydro-2′H-spiro[imidazolidine-4,1′-naphthalene]-2,5-dione (**3**)

Creamy powder. Yield: *60*%; mp 168.8–170.2 °C; TLC: *R_f_* = *0.71 (S_3_)*; HPLC: *t*_R_ = *1.011*; LC/MS: *C_28_H_34_N_4_O_3_* (*98*%) *m/z*: 474.59, found: *475.34*; ^1^H NMR (500 MHz, CDCl_3_-*d*) δ (ppm): 1.53–1.62 (m, 2 H, CH_2_C*H*_2_(CH_2_)_2_) 1.68–1.75 (m, 2 H, (CH_2_)_2_C*H*_2_CH_2_) 1.76–1.84 (m, 1 H, tetraline) 1.93–2.00 (m, 1 H, tetraline) 2.21–2.34 (m, 2 H, tetraline) 2.45 (br. s., 2 H, (C*H*_2_)_3_C*H*_2_) 2.49 (s, 3 H, C*H*_3_) 2.55–2.63 (m, 4 H, N_1pip_(C*H*_2_)_2_) 2.79–2.90 (m, 2 H, tetraline) 3.34 (br. s., 4 H, N_4pip_(C*H*_2_)_2_) 3.58 (t, *J* = 7.16 Hz, 2 H, C*H*_2_(CH_2_)_3_) 6.04 (br. s., 1 H, N_1_*H*_hyd_) 6.81–6.86 (m, 2 H, Ar) 7.03 (d, *J* = 8.02 Hz, 1 H, Ar) 7.11–7.23 (m, 3 H, Ar) 7.82–7.87 (m, 2 H, Ar). ^13^C NMR (126 MHz, CDCl_3_-*d*) δ (ppm): 19.13, 23.91, 26.18, 26.20, 28.88, 34.27, 38.60, 47.30, 52.86, 57.92, 62.75, 113.42, 126.47, 126.95, 127.59, 128.77, 129.90, 130.46, 133.11, 138.24, 154.24, 156.87, 176.32, 196.71.

#### 2.2.4. (R,S)-1-(5-(4-(4-Acetylphenyl)piperazin-1-yl)pentyl)-3′,4′-dihydro-2′H-spiro[imidazolidine-4,1′-naphthalene]-2,5-dione (**4**)

Creamy powder. Yield: *75*%; mp 134.3–135.1 °C; TLC: *R_f_* = *0.73 (S_3_)*; HPLC: *t*_R_ = *1.031*; LC/MS: *C_29_H_36_N_4_O_3_* (99%) *m/z*: 488.62, found: *489.37*; ^1^H NMR (500 MHz, CDCl_3_-*d*) δ (ppm): 1.31–1.39 (m, 2 H, (CH_2_)_2_C*H*_2_(CH_2_)_2_) 1.55 (quin, *J* = 7.45 Hz, 2 H, (CH_2_)_3_C*H*_2_CH_2_) 1.68 (quin, *J* = 7.45 Hz, 2 H, CH_2_C*H*_2_(CH_2_)_3_) 1.78 (td, *J* = 8.02, 5.16 Hz, 1 H, tetraline) 1.91–1.97 (m, 1 H, tetraline) 2.19–2.30 (m, 2 H, tetraline) 2.34–2.39 (m, 2 H, (CH_2_)_4_C*H*_2_) 2.48 (s, 3 H, C*H*_3_) 2.53–2.58 (m, 4 H, N_1pip_(C*H*_2_)_2_) 2.76–2.89 (m, 2 H, tetraline) 3.29–3.34 (m, 4 H, N_4pip_(C*H*_2_)_2_) 3.54 (t, *J* = 7.16 Hz, 2 H, C*H*_2_(CH_2_)_4_) 6.36 (br. s, 1 H, N_1_*H*_hyd_) 6.82 (d, *J* = 9.17 Hz, 2 H, Ar) 7.00 (d, *J* = 7.45 Hz, 1 H, Ar) 7.08–7.20 (m, 3 H, Ar) 7.83 (d, *J* = 8.59 Hz, 2 H, Ar). ^13^C NMR (126 MHz, CDCL_3_-*d*) δ (ppm): 19.13, 24.66, 26.19, 26.22, 28.06, 28.89, 34.26, 38.61, 47.25, 52.88, 58.41, 62.72, 113.42, 126.48, 126.93, 127.59, 128.73, 129.87, 130.46, 133.18, 138.22, 154.21, 156.95, 176.34, 196.69.

#### 2.2.5. (R,S)-1-{4-[4-(4-Acetylophenyl)piperazyn-1-yl]butyl}-1′,3′-dihydro-2H,5H-spiro[imidazolidine-4,2′-indene]-2,5-dione (**5**)

Creamy powder. Yield: *62*%; mp *213.8*–*214.9* °C; TLC: *R_f_* = *0.28 (S_4_)*; HPLC: *t*_R_ = *1.157*; LC/MS: *C_27_H_32_N_4_O_3_* (100%) *m/z*: *460.57*, found: *461.38*; ^1^H NMR (300 MHz, CDCl_3_-*d*) δ (ppm): 1.55 (quin, *J* = 7.59 Hz, 2 H, CH_2_C*H*_2_(CH_2_)_2_) 1.67–1.75 (m, 2 H, (CH_2_)_2_C*H*_2_CH_2_) 2.41 (t, *J* = 7.30 Hz, (CH_2_)_3_C*H*_2_) 2.50 (s, 3 H, C*H*_3_) 2.53–2.60 (m, 4 H, N_1pip_(C*H*_2_)_2_) 3.02 (s, 1 H, indane) 3.06 (s, 1 H, indane) 3.30–3.38 (m, 4 H, N_4pip_(C*H*_2_)_2_) 3.58 (t, *J* = 7.30 Hz, 2 H, C*H*_2_(CH_2_)_3_), 3.61 (s, 1 H, indane) 3.64 (s, 1 H, indane) 5.84 (br.s, 1 H, N_1_*H*_hyd_) 6.85 (d, *J* = 9.16 Hz, 2 H, Ar) 7.22 (s, 4 H, Ar) 7.86 (d, *J* = 9.16 Hz, 2 H, Ar). ^13^C NMR (126 MHz, CDCL_3_-*d*) δ (ppm): 23.83, 26.13, 26.18, 38.63, 44.28, 47.17, 52.84, 57.97, 68.35, 68.46, 113.44, 124.64, 127.50, 127.54, 130.52, 138.98, 154.25, 156.67, 176.27, 197.15.

#### 2.2.6. (R,S)-1-{5-[4-(4-Acetylophenyl)piperazyn-1-yl]pentyl}-1′,3′-dihydro-2H,5H-spiro[imidazolidine-4,2′-indene]-2,5-dione (**6**)

Creamy powder. Yield: *86*%; mp *157.4*–*157.7* °C; TLC: *R_f_* = *0.43 (S_1_)*; HPLC: *t*_R_ = *1.197*; LC/MS: *C_28_H_34_N_4_O_3_* (*99*%) *m/z*: *474.59*, found: *475.41*; ^1^H NMR (300 MHz, CDCl_3_-*d*) δ (ppm): 1.31–1.39 (m, 2 H, (CH_2_)_2_C*H*_2_(CH_2_)_2_) 1.51–1.59 (m, 2 H, (CH_2_)_3_C*H*_2_CH_2_) 1.67 (quin, *J* = 7.45 Hz, 2 H, CH_2_C*H*_2_(CH_2_)_3_) 2.36 (t, *J* = 7.59 Hz, 2 H, (CH_2_)_4_C*H*_2_) 2.49 (s, 3H, C*H*_3_) 2.55 (d, *J* = 4.01 Hz, 4 H, N_1pip_(C*H*_2_)_2_) 3.01 (s, 1 H, indane) 3.04 (s, 1 H, indane) 3.33 (d, *J* = 3.72 Hz, 4 H, N_4pip_(C*H*_2_)_2_) 3.53 (t, *J* = 7.30 Hz, 2 H, C*H*_2_(CH_2_)_4_) 3.58 (s, 1 H indane) 3.61 (m, 1 H, indane) 6.17 (br. s., 1 H, N_1_*H*_hyd_) 6.84 (d, *J* = 7.45 Hz, 2 H, Ar) 7.20 (s, 4 H, Ar) 7.84 (d, *J* = 8.88 Hz, 2 H, Ar). ^13^C NMR (126 MHz, CDCL_3_-*d*) δ (ppm): 24.68, 26.19, 26.23, 28.08, 38.74, 44.39, 47.26, 52.85, 58.37, 68.36, 68.39, 113.42, 113.76, 124.69, 127.58, 130.47, 130.90, 139.04, 154.22, 156.49, 176.10, 196,74.

#### 2.2.7. (R,S)-1-{4-[4-(4-Acetylophenyl)piperazyn-1-yl]butyl}-3′,4′-dihydro-2H,2′H,5H-spiro[imidazolidine-4,1′-naftalene]-2,5-dione (**7**)

Creamy powder. Yield: *47*%; mp *199.3*–*199.6* °C; TLC: *R_f_* = *0.42 (S_1_)*; HPLC: *t*_R_ = *1.234*; LC/MS: *C_28_H_34_N_4_O_3_* (*96*%) *m/z*: *474.59*, found: *475.34*; ^1^H NMR (300 MHz, CDCl_3_-*d*) δ (ppm): 1.55 (d, *J* = 5.44 Hz, 2 H, CH_2_C*H*_2_(CH_2_)_2_) 1.65–1.74 (m, 2 H, (CH_2_)_2_C*H*_2_CH_2_) 1.83–1.91 (m, 1 H, tetraline) 2.16–2.28 (m, 1 H, tetraline) 2.41 (t, *J* = 5.16 Hz, 2 H, (C*H*_2_)_3_C*H*_2_) 2.50 (s, 3 H, C*H*_3_) 2.56 (br. s., 4 H, N_1pip_(C*H*_2_)_2_) 2.71 (dd, *J* = 16.32, 1.43 Hz, 1 H, tetraline) 2.89 (ddd, *J* = 17.69, 11.81, 6.30 Hz, 1 H, tetraline) 2.99–3.07 (m, 1 H, tetraline) 3.31–3.40 (m, 5 H, N_4pip_(C*H*_2_)_2_, tetraline) 3.51–3.59 (m, 2 H, C*H*_2_(CH_2_)_3_) 5.70 (br. s, 1 H, N_1_*H*_hyd_) 6.82–6.90 (m, 2 H, Ar) 7.03–7.19 (m, 4 H, Ar) 7.81–7.89 (m, 2 H, Ar). ^13^C NMR (126 MHz, CDCL_3_-*d*) δ (ppm): 25.00, 26.17, 26.20, 30.17, 37.57, 38.57, 47.35, 52.92, 57.98, 60.60, 113.43, 126.71, 127.02, 127.63, 129.08, 129.68, 130.47, 131.78, 154.24, 156.52, 176.50, 196.67.

#### 2.2.8. (R,S)-1-{5-[4-(4-Acetylophenyl)piperazin-1-yl]pentyl}-3′,4′-dihydro-2H,2′H,5H-spiro[imidazolidine-4,1′-naftalene]-2,5-dione (**8**)

Creamy powder. Yield: *82*%; mp *178.7*–*179.6* °C; TLC: *R_f_* = *0.66 (S_1_)*; HPLC: *t*_R_ = *1.273*; LC/MS: *C_29_H_36_N_4_O_3_* (*99*%) *m/z*: *488.62*, found: *489.37*; ^1^H NMR (300 MHz, CDCl_3_-*d*) δ (ppm): 1.36 (quin, *J* = 7.73 Hz, 2 H, (CH_2_)_2_C*H*_2_(CH_2_)_2_) 1.56 (dt, *J* = 15.18, 7.59 Hz, 2 H, (CH_2_)_3_C*H*_2_CH_2_) 1.67 (dt, *J* = 14.96, 7.55 Hz, 2 H, CH_2_C*H*_2_(CH_2_)_3_) 1.87 (dd, *J* = 13.32, 6.16 Hz, 1 H, tetraline) 2.18–2. 27 (m, 1 H, tetraline) 2.35–2.40 (m, 2 H, (CH_2_)_4_C*H*_2_) 2.50 (s, 3 H, C*H*_3_) 2.53–2.59 (m, 4 H, N_1pip_(C*H*_2_)_2_) 2.71 (dd, *J* = 16.47, 2.15 Hz, 1 H, tetraline) 2.85–2.94 (m, 1 H, tetraline) 3.00–3.06 (m, 1 H, tetraline) 3.32–3.39 (m, 5 H, tetraline, N_4pip_(C*H*_2_)_2_) 3.53 (t, *J* = 7.30 Hz, 2 H, C*H*_2_(CH_2_)_4_) 5.63 (br. s, 1 H, N_1_*H*_hyd_) 6.81–6.87 (m, 2 H, Ar) 7.04 (d, *J* = 7.45 Hz, 1 H, Ar) 7.10–7.19 (m, 3 H, Ar) 7.82–7.88 (m, 2 H, Ar). ^13^C NMR (126 MHz, CDCL_3_-*d*) δ (ppm): 24.65, 25.00, 26.20, 26.34, 28.04, 30.17, 37.56, 38.60, 47.35, 52.94, 58.41, 60.58, 113.41, 126.68, 126.98, 127.59, 129.08, 129.65, 130.47, 131.81, 133.92, 154.25, 156.58, 176.53, 196.68.

### 2.3. General Procedure for Obtaining the Final Compounds ***9***–***11***

7-Ketonyl derivatives of 8-bromo-1,3-dimethyl-3,7-dihydro-1H-purine-2,6-dione XIII, XIV (5 mmol) [21] with 4′-(1-piperazinyl)acetophenone (10 mmol) were refluxed in 2-methoxyethanol (20 mL) for 12h. After concentration of the reaction mixtures, the resulting residues were purified by flash column chromatography.

#### 2.3.1. 8-(4-(4-(4-Acetylphenyl)piperazin-1-yl)butyl)-1,3-dimethyl-1H-imidazo[2,1-f]purine-2,4(3H,8H)-dione (**9**)

Creamy solid. Yield: *90%*; mp 252–253^o^C; TLC: *R_f_* = *0.*59 (S*_4_*); HPLC: *t_R_* = 0.996; LC/MS: *C_25_H_31_N_7_O_3_* (96%) *m/z*: 477.55, found 478.50; ^1^H-NMR (CDCl_3_) *δ* (ppm): 1.25–1.26 (m, 2H, CH_2_CH_2_C*H*_2_CH_2_), 2.29–2.33 (m, 2H, CH_2_CH_2_CH_2_C*H*_2_), 2.45–2.61 (m, 7H, N_1pip_(C*H*_2_)_2_ + C*H*_3_), 3.28–3.60 (m, 12H, N_1_-C*H*_3_ + N_3_-C*H*_3_ + CH_2_C*H*_2_CH_2_CH_2_+ N_4pip_(C*H*_2_)_2_),4.10 (t, 2H, *J* = 7.1 Hz, N_8_C*H*_2_), 6.79–6.89 (m, 3H, Ar, C_6_*H*), 7.42 (d, 1H, *J* = 2.3 Hz, C_7_*H*), 7.74–7.90 (m, 2H, Ar). ^13^C NMR (126 MHz, CDCL_3_-*d*) δ (ppm): 21.73, 23.12, 25.97, 27.31, 27.82, 30.08, 45.69, 46.72, 48.94 (t, 3C), 52.58, 57.46, 100.07, 107.82, 113.75, 118.70, 127.56, 130.53, 147.41, 151.96, 152.21, 154.01, 154.23, 196.74

#### 2.3.2. 8-(4-(4-(4-Acetylphenyl)piperazin-1-yl)butyl)-1,3,7-trimethyl-1H-imidazo[2,1-f]purine-2,4(3H,8H)-dione (**10**)

Yellow oil. Yield: 75*%*; TLC: *R_f_* = *0.*66 (S*_4_*); HPLC: *t_R_* = 1.030; LC/MS: *C_26_H_33_N_7_O_3_* (97%) *m/z*: 491.58, found 492.50; ^1^H-NMR (CDCl_3_) *δ* (ppm): 1.25–1.26 (m, 2H, CH_2_CH_2_C*H*_2_CH_2_), 2.01–2.06 (m, 2H, CH_2_CH_2_CH_2_C*H*_2_), 2.50–2.61 (m, 7H, N_1pip_(C*H*_2_)_2_ + C*H*_3_), 3.28–3.60 (m, 12H, N_1_-C*H*_3_ + N_3_-C*H*_3_ + CH_2_C*H*_2_CH_2_CH_2_+ N_4pip_(C*H*_2_)_2_), 4.05 (t, 2H, *J* = 7.1 Hz, N_8_C*H*_2_), 6.84–6.92 (m, 2H,Ar), 7.17–7.21 (m, 1H,Ar), 7.84–7.88 (m, 2H, Ar + C_6_*H*). ^13^C NMR (126 MHz, CDCL_3_-*d*) δ (ppm): 10.54, 23.79, 26.01, 26.23, 27.39, 27.88, 30.19, 33.58, 41.31, 43.23, 47.35, 52.91, 57.64, 93.39, 100.0, 107.74, 105.71, 113.46, 126.87, 127.76, 147.64, 151.50, 152.10, 154.12, 191.83.

#### 2.3.3. 8-(5-(4-(4-Acetylphenyl)piperazin-1-yl)pentyl)-1,3-dimethyl-1H-imidazo[2,1-f]purine-2,4(3H,8H)-dione (**11**)

Yellow oil. Yield: 84*%*;TLC: *R_f_* = *0.*68 (S*_4_*); HPLC: *t_R_* = 1.038; LC/MS: *C_26_H_33_N_7_O_3_* (97%) *m/z*: 477.55, found 478.50; ^1^H-NMR (CDCl_3_) *δ* (ppm): 2.32–2.44 (m, 9H, CH_2_CH_2_C*H*_2_C*H*_2_C*H*_2_, C*H*_3_), 3.35–3.46 (m, 9H, N_1pip_(C*H*_2_)_2_), N_4pip_(C*H*_2_)_2_, N_3_-C*H*_3_), 3.57 (s, 7H, CH_2_C*H*_2_CH_2_CH_2_CH_2_, N_1pip_(C*H*_2_)_2_), N_1_-C*H*_3_), 4.07 (t, 2H, *J* = 7.1 Hz, N_8_C*H*_2_), 6.78–6.84 (m, 3H, Ar), 7.40–7.46 (m, 1H, Ar), 7.85–7.87 (d, 2H, *J* = 8.8 Hz C_7_*H* + C_6_*H*). ^13^C NMR (126 MHz, CDCL_3_-*d*) δ (ppm): 23.79, 26.23, 27.39, 27.88, 30.19, 33.58, 41.31, 43.23, 47.35, 52.91, 57.64, 93.39, 100.0, 107.74, 105.71, 113.46, 126.87, 127.76, 147.64, 151.50, 152.10, 154.12, 191.83.

### 2.4. General Procedure for Obtaining the Final Compounds ***12***–***14***

The starting 9-chloroalkyl-1,3-dimethyl-pirymido[2,1-*f*]purine-2,4,8-(1H,3H,9H)-triones (**XV**–**XVII**) were obtained according to the previously described procedure [22]. Mixtures of **XV**–**XVII** (5 mmol) with a two-fold excess of 4′-(1-piperazinyl)acetophenone (10 mmol) in acetonitrile (4 mL) were exposed to microwave irradiation in a MW oven for 1 h at a power of 200 W. After evaporation of the solvent, the products were purified by flash column chromatography on silica gel using appropriate eluent systems.

#### 2.4.1. 9-(4-(4-(4-Acetylphenyl)piperazin-1-yl)butyl)-1,3-dimethylpyrimido[2,1-f]purine-2,4,8(1H,3H,9H)-trione (**12**)

Yellow oil. Yield: 78*%*;TLC: *R_f_* = *0.*71 (S*_4_*); HPLC: *t_R_* = 1.020; LC/MS: *C_26_H_31_N_7_O_3_* (96%) *m/z*: 505.56, found 506.32; ^1^H-NMR (CDCl_3_) *δ* (ppm): 1.24–1.28 (m, 4H, CH_2_*CH_2_CH_2_*CH_2_), 2.44–2.51 (m, 9H, CH_2_CH_2_CH_2_C*H*_2_ + C*H*_3_^+^ N_1pip_(C*H*_2_)_2_), 3.33–3.43 (m, 7H, N_4pip_(C*H*_2_)_2_) + N_3_-C*H*_3_), 3.61 (s, 3H, N_1_-C*H*_3_), 4.31 (t, *J* = 6.4 Hz, 2H, CH_2_CH_2_CH*_2_*C*H*_2_), 6.26–6.31 (d, *J* = 7.1, 1H, C_6_*H*), 6.83–6.91 (m, 2H, Ar), 7.84–7.89 (m, 2H, Ar), 8.50–8.53 (d, *J* = 8.1 Hz, 1H, C_7_*H*). ^13^C NMR (126 MHz, CDCL_3_-*d*) δ (ppm): 14.22, 22.78, 25.00, 25.36, 28.01, 29.45, 29.69, 29.71, 30.32, 32.01, 43.26, 47.35, 52.93, 57.96, 100.83, 107.98, 113.47, 130.45, 133.33, 137.41, 147.35, 149.83, 151.61, 154.18, 196.63.

#### 2.4.2. 9-(5-(4-(4-Acetylphenyl)piperazin-1-yl)pentyl)-1,3-dimethylpyrimido[2,1-f]purine-2,4,8(1H,3H,9H)-trione (**13**)

Yellow oil. Yield: 82*%*;TLC: *R_f_* = *0.*68 (S*_4_*); HPLC: *t_R_* = 1.080; LC/MS: *C_27_H_33_N_7_O_4_* (96%) *m/z*: 505.56, found 506.32; ^1^H-NMR (CDCl_3_) *δ* (ppm): 0.99–1.44 (m, 4H, CH_2_CH_2_*C*H*_2_CH_2_*C*H*_2_), 1.49–1.59 (m, 2H, CH_2_CH_2_*CH_2_C*H*_2_*CH_2_), 1.64–1.84 (m, 2H, CH_2_C*H*_2_*C*H*_2_C*H*_2_*CH_2_), 2.38–2.71 (m, 7H, C*H*_3_ + N_1pip_(C*H*_2_)_2_), 3.31–3.44 (m, 5H, N_4pip_(C*H*_2_)_2_ + N_3_-C*H*_3_), 3.61–3.59 (m, 3H, N_1_-C*H*_3_), 4.23–4.29 (m, 2H, CH_2_CH_2_CH_2_CH*_2_*C*H*_2_), 6.26–6.29 (m, 1H, Ar), 6.60–6.64 (m, 1H, C_6_*H*), 6.79–6.85 (m, 1H, Ar), 7.79–7.88 (m, 2H, Ar), 8.48–8.51 (d, *J* = 8.1 Hz, 1H, C_7_*H*). ^13^C NMR (126 MHz, CDCL_3_-*d*) δ (ppm): 23.91, 24.66, 26.21, 28.01, 28.68, 29.45, 29.69, 29.99, 30.32, 43.31, 47.22, 52.67, 58.31, 67.93, 100.81, 101.99, 107.96, 113.50, 113.50, 133.80, 135.86, 147.35, 149.84, 151.50, 154.17, 159.04, 196.60.

#### 2.4.3. 9-(4-(4-(4-Acetylphenyl)piperazin-1-yl)butyl)-7-bromo-1,3-dimethylpyrimido[2,1-f]purine-2,4,8(1H,3H,9H)-trione (**14**)

Yellow-cream oil. Yield: 92*%*;TLC: *R_f_* = *0.*79 (S*_4_*); HPLC: *t_R_* = 1.31; LC/MS: *C_26_H_30_BrN_7_O_4_* (96%) *m/z*: 584.46, found 586.23; ^1^H-NMR (CDCl_3_) *δ* (ppm): 1.20–1.33 (m, 3H, CH_2_*CH_2_CH_2_*CH_2_), 1.82–1.92 (m, 3H, CH_2_*C*H*_2_CH_2_*C*H*_2_), 2.44–2.51 (m, 8H, CH_2_CH_2_CH_2_C*H*_2_ + C*H*_3_ + N_1pip_(C*H*_2_)_2_), 3.33–3.43 (m, 6H, N_4pip_(C*H*_2_)_2_) + N_3_-C*H*_3_), 3.61 (s, 3H, N_1_-C*H*_3_), 4.31 (t, *J* = 6.4 Hz, 2H, CH_2_CH_2_CH*_2_*C*H*_2_), 6.28–6.31 (m, 1H, Ar), 6.83–6.91 (m, 2H, Ar), 7.84–7.89 (m, 1H, Ar), 8.50–8.53 (d, *J* = 8.1 Hz, 1H, C_6_*H*). ^13^C NMR (126 MHz, CDCL_3_-*d*) δ (ppm): 19.71, 24.51, 26.24, 28.07, 28.68, 29.45, 30.32, 30.40, 31.71, 44.80, 46.99, 52.66, 58.06, 100.51, 104.55, 113.69, 114.48, 128.08, 130.47, 133.68, 146.54, 149.61, 151.50, 153.91, 155.78, 196.64.

### 2.5. Cell Culture Condition for MTT Assay

Primary and metastatic colon cancer (SW480, SW620), metastatic prostate cancer (PC3), and human immortal keratinocyte (HaCaT) cell lines and immortal microvascular endothelial cells (HMEC-1) were obtained from the American Type Culture Collection (ATCC, Rockville, USA) and cultured in recommended medium, namely, MEM (Biowest SAS, France) for the SW480 and SW620 cells, RPMI 1640 (Biowest SAS, Nuaillé, France) for PC3, DMEM (Biowest SAS, France) for HaCaT and MCDB 131 (PAN BioTech, Aidenbach, Germany) for HMEC-1. The SW480 and SW620 cell lines were initiated by A. Leibovitz et al. [23] and derived from a human primary colon adenocarcinoma and lymph node metastasis of the same patient. respectively. PC3 cell cells were initiated from a bone metastasis of a human prostatic adenocarcinoma. HaCaT cells were obtained from adult human skin, while HMEC-1 cells were established from the dermis of a human neonate. Cells were cultured in recommended medium supplemented with 10% FBS (Biowest SAS, Nuaillé, France), penicillin (100 U/mL), streptomycin (100 mg/mL) (Gibco Life Technologies, Waltham MA, USA) and incubated at 37 °C under 5% CO_2_. The culture medium of HMEC-1 cells was additionally supplemented with glutamine (1 mM) and microvascular growth supplement (5%) (MVGS; Gibco, USA). The cells were cultured until 80% confluency was reached, harvested by treatment with 0.25% trypsin 0.02% EDTA (Gibco Life Technologies, Waltham, MA, USA) and subsequently used for further experiments.

### 2.6. MTT Assay

Cell viability was estimated using a conversion of 3-(4,5-dimethylthiazol-2-yl)-2,5-diphenyltetrazolium bromide salt (MTT) to insoluble formazan crystals by mitochondrial dehydrogenases, present in living cells. Cells were cultured in 96-well plates (l × 10^4^ cells per well) and incubated for 24 h at 37 °C under 5% CO_2_. Next, the cells were treated with various concentrations of the tested compounds (ranging from 10 to 180 μM) and incubated for 72 h at 37 °C under 5% CO_2_. Untreated cells were used as the control. Next, the medium was removed, MTT solution (0.5 mg/mL) was added to each well, and the cells were incubated for 4 h at 37 °C under 5% CO_2_. After that, the obtained formazan crystals were dissolved in DMSO and isopropanol (1:1, *v/v*). The optical density of the dissolved crystals was measured at a wavelength of 570 nm using a UVM 340 reader (ASYS Hitech GmbH, Austria). Cell viability was presented as a percent of MTT reduced in treated cells versus control cells. The relative MTT level (%) was calculated as [A]/[B] × 100, where [A] is the absorbance of the test sample, and [B] is the absorbance of the control sample. The IC_50_ value was estimated using CompuSyn version 1.0.

### 2.7. Determination of Hemolytic Activity

The hemolysis experiment was performed according to the previous report [24] and was approved by the Bioethics Commission at the Lower Silesian Medical Chamber (1/PNHAB/2018). Freshly isolated human erythrocytes were incubated with compound **4** dissolved in DMSO (final concentration of 10^−6^ or 10^−5^ M) in PBS buffer at 37 °C for 30 min. The samples were then centrifuged and the absorbance of hemoglobin in the supernatants was measured at 540 nm. Additionally, negative (erythrocytes in PBS buffer), positive (erythrocytes in distilled water), and DMSO controls were also performed.

### 2.8. Thymidine Phosphorylase Inhibition

TP/PD-ECGF (*E. coli* TP (Sigma T6632)) activity was determined by measuring the absorbance at 290 nm spectrophotometrically, according to the manufacturer′s procedure [25]. Briefly the reaction mixture of 145 µL of potassium phosphate buffer (pH 7.4), 20 µL of 1.5 mM Thymidine 50 monophosphate solution as substrate, and 30 µL of an enzyme (*E. coli* TP, 0.05 and 0.002 U) was incubated with 5 µL of test materials for 10 min at 25 °C in a 96-well, flat bottom microplate (200 µL). Readings were taking by a microplate reader (SpectraMax Plus384, USA) at 290 nm. The readings were taken continuously after 10, 20, and 30 min by a microplate reader. All assays were performed in triplicate.

### 2.9. Docking Studies

Compound **4** was prepared in Corina online. Isomers were generated in Maestro. Atom types were checked, and charges and hydrogens were added in Sybyl 8.0 (Tripos). Prepared ligands were saved in mol2 format. For docking studies, the crystal structure of thymidine phosphorylase from *E. coli* was used (PDB code: 4EAD) [26,27]. The structure of the enzyme was obtained with a high resolution of 1.5 Å in a complex with ONP (3′-azido-2′-fluoro-dideoxyuridine) as the ligand. Protein preparation was carried out using an earlier validated procedure [28]. For docking, sulfate was replaced by a dihydrogenphosphate ion [29]. Next, hydrogen atoms were added, all histidines were protonated at Nε, and the ligand was removed. The binding site was defined as residues within 10 Å from the ONP ligand. During the docking, water molecules within a distance of 5 Å from the ONP ligand were taken into account with the ‘toggle’ option. The calculation was performed using the Gold 5.1 program with standard settings, and a genetic algorithm with a population size of 100 and 100,000 number of operations. For each ligand, 10 poses were obtained and sorted with a GoldScore value. Results were analyzed in PyMOL and Maestro.

## 3. Results

### 3.1. Chemistry

**Scheme 1:**
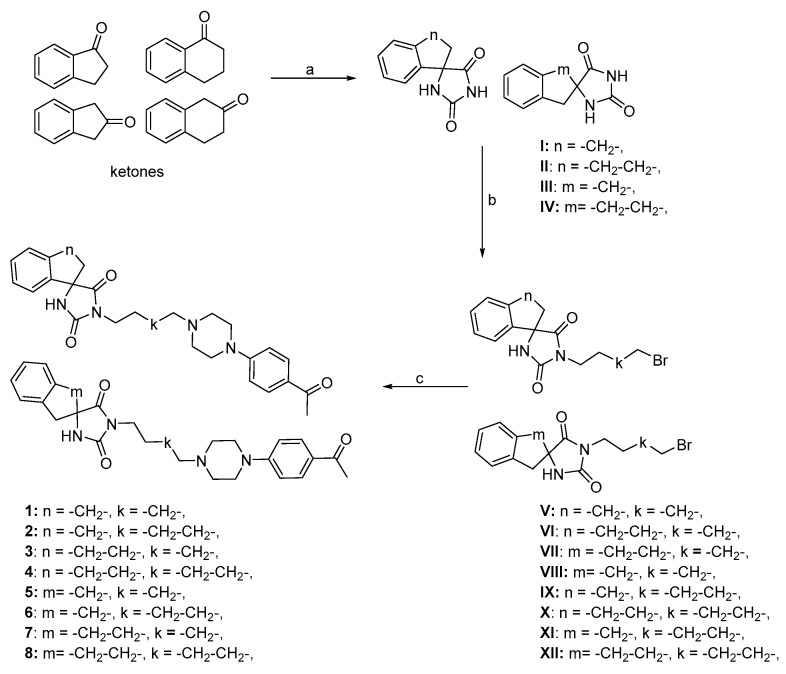
Reagents and conditions: (**a**) KCN, (NH_4_)_2_CO_3_, 50% EtOH, 60 °C, 16 h; (**b**) 1,4-dibromobutane or 1.5-dibromopentane, K_2_CO_3_, MeCN, 80 °C, 30 h; (**c**) 4′-(1-piperazinyl)acetophenone, K_2_CO_3_, Ac, 60 °C, 20 h.

**Scheme 2:**
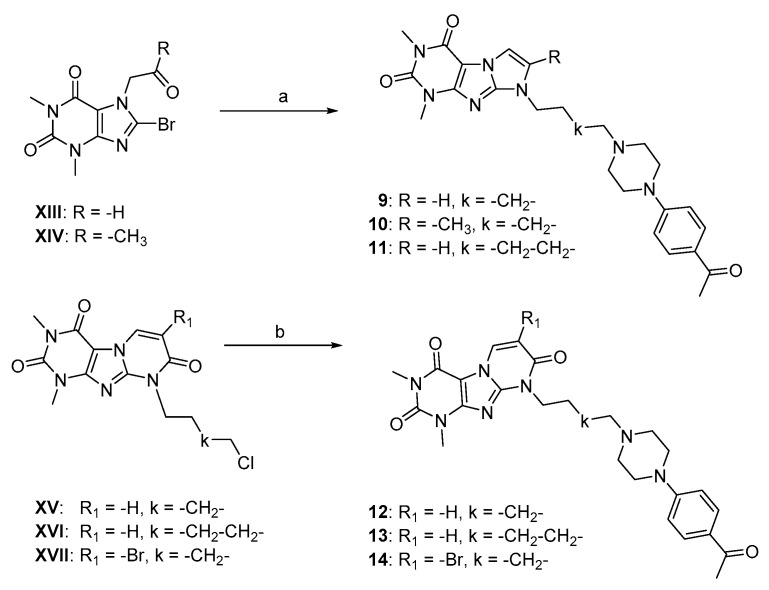
Reagents and conditions: (**a**) N-(aminoalkyl)-4-acetylphenylpiperazines, 2-methoxyethanol, reflux, 12 h; (**b**) 9-(chloroalkyl)-purine derivative, 4′-(1-piperazinyl)acetophenone, MeCN, 80 °C, 1 h, MW (200 W).

The series of designed spirohydantoin, imidazo- and pyrimidino[2,1-f]purine derivatives (**1**–**14**) were obtained in multistep synthesis pathways in moderate to good yields (47–92%), as shown in Scheme 1 and Scheme 2.

Synthetic routes and chemical structures of the designed spirohydantoin derivatives (**1**–**8**) are depicted in Scheme 1. Spirohydantoin rings (**I**–**IV**) were synthesized from commercially available ketones in the Bucherer–Berg reaction. This starting spirohydantoin moiety was coupled with 1,4-dibromobutane or 1,5-dibromopentane to give alkylated intermediate compounds **V**–**XII**. Final compounds (**1**–**8**) were obtained through coupling intermediate **V**–**XII** with 4′-(1-piperazinyl)acetophenone.

Synthetic routes and chemical structures of the designed 1,3-dimethyl-(1H,8H)-imidazo[2,1-*f*]purine-2,4-dione derivatives (**9**–**11**) and pyrimido[2,1-*f*]purine-2,4,8-trione (**12**–**14**) are depicted in Scheme 2. Compounds **9**–**11** were obtained from a cyclocondensation reaction of 7-ketonyl derivatives of 8-bromotheophylline **XIII**–**XIV** with the appropriate *N*-(aminoalkyl)-4-acetylphenylpiperazines, according to the previously described procedures [21]. Compounds **12**–**14** were obtained after microwave irradiation of 9-(chloroalkyl)-1,3-dimethyl-(1H,3H,9H)-pyrimido[2,1-*f*]purine-2,4,8-triones (**XV**,**XVI**) with a two-fold excess of 4′-(1-piperazinyl)acetophenone, in acetonitrile (MeCN) for 1 h [22].

The final compounds were isolated from the reaction mixture and further purified by column chromatography using appropriate eluent systems. The purity of the final compounds (**1**–**14**) was above 95%. The structures of all final compounds were assessed based on chromatography (HPLC, LC/MS) and spectral (^1^HNMR, ^13^CNMR,) analysis. For further in vitro studies compounds were used as free bases.

### 3.2. Biological Study

To assess the cytotoxicity of the compounds, their in vitro antiproliferative activity against prostate and colon human cancer cell lines and non-cancerous cell lines was determined by the MTT method (Table 1).

Based on the obtained IC_50_ values, a spectrum of cytotoxic activities was observed for the synthesized compounds, **1**–**14**. Compounds **1**, **2**, **4**, **6**, and **8** were effective against human primary colon cancer SW480 cells, and among them, compound **4** was the most effective with an IC_50_ = 16.8 µM. Compounds **9**–**14** exhibited no activity against this cell line. In contrast, the effectiveness of the compounds against human metastatic colon cancer SW620 cells ranged from moderate to lack of it. However, compound **4** was active, with an IC_50_ = 12.9 µM against this cell line. The tested compounds had moderate to weak activity against the PC3 cancer cell line. Again, compound **4** was active with an IC_50_ = 20.58 µM. Also, a 72-h exposure of normal human keratinocytes (HaCaT) to compound **4**, as a control, revealed that this compound had no significant cytotoxic effect on these cells (Table 1).

To evaluate tumor-targeting selectivity, the most active compound, compound **4,** was additionally tested against the immortal human microvascular endothelial cell line, HMEC-1. The obtained IC_50_ value (149.29 µM) was much higher, as compared to all the tested cancer cells, with favorable SI parameters (8.88 for SW480, 11.57 for SW620, and 7.25 for PC3 cells) (Table 2).

### 3.3. Hemolytic Activity

Our further research focused on the determination of the hemolytic activity of compound **4** against human red blood cells. A hemolysis test revealed that this compound had no toxicity to erythrocytes at concentrations of 10^−6^ or 10^−5^ M, as no hemolytic activity was detected.

### 3.4. Thymidine Phosphorylase Inhibition Assay

Compound **4** was evaluated as a TP inhibitor. The tested ligand displayed only a 21.21% inhibition value of thymidine phosphorylase activity at a 100 µM screening concentration. For comparison, 7-DX used as a reference inhibitor, showed 54.55% inhibition at the same concentration.

### 3.5. Docking Studies to the Thymidine Phosphorylase Active Site

**Figure 4:**
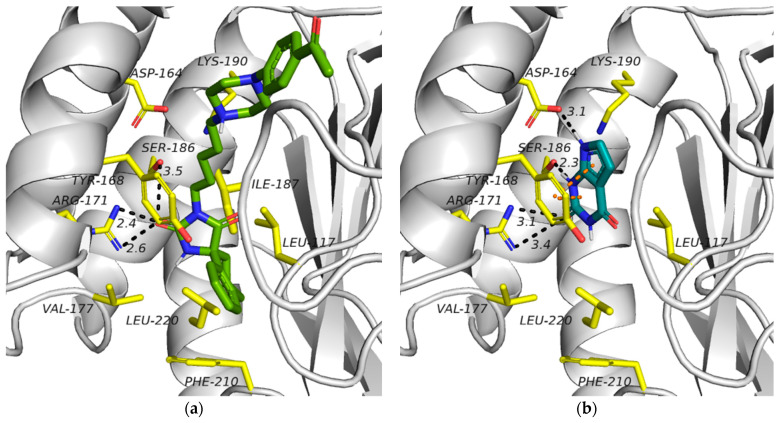
Binding mode of the *R*-isomer of compound **4** (**a**) and 7-DX (**b**) within the active site of TP. Amino acid residues colored in yellow. Hydrogen bonds and π–π stacking interactions are presented as black and orange dashes, respectively.

Molecular modeling studies were applied to describe the binding mode of the compound *R*-isomer of compound **4** within the catalytic cleft of thymidine phosphorylase (Figure 4).

## 4. Discussion

Cancer is a serious global problem, being one of the most lethal human diseases. Difficulties in diagnosis, recurrence, and resistance to the currently approved chemotherapies still necessitate the discovery of new, effective, and safe drugs. In this study, we tested the antiproliferative effect of two groups of compounds against different human cancer cell lines, namely prostate (PC3) and colon (SW480, SW620).

The analysis of structure-activity relationships revealed that the growth-inhibitory potency of compounds **1**–**14** depended mainly on the structure of the terminal cyclic amide/imide moieties. A moderate growth-inhibitory potency against PC3 was observed for compounds **2** and **8**, in which the hydantoin scaffold was substituted by an indane or tetralin moiety. The highest growth-inhibitory potencies for a panel of different human cancer cell lines were indicated for compound **4**, a derivative of 3′,4′-dihydro-2′H-spiro[imidazolidine-4,1′-naphthalene]-2,5-dione. Its high tumor-targeting selectivity indicates that compound **4** exhibits no toxic effect on control, non-cancer cells. These promising results form the basis for future research, including in vivo studies, with the use of xenograft models including patient-derived cancer cells. This observation inspired us to further evaluate the hemolytic activity of compound **4**. Determination of hemolysis is a very important assay to predict in vivo toxicity as, during entry to the body compound **4** will have contact with erythrocytes. The obtained results confirmed no toxicity to red blood cells. This is in good agreement with the results reported by Su et al. [30], who showed that other hydantoin derivatives have also only limited hemolytic activity.

The results of the thymidine phosphorylase inhibitory activity assay showed that the growth inhibitory properties of compound **4** are weakly associated with thymidine phosphorylase inhibition. This was surprising, as the compound seemed to meet all the criteria for potential TP inhibition, such as a commitment to creating hydrogen bonds. Molecular modeling studies described the binding mode of compound **4** within the catalytic cleft of TP (Figure 4a), compared to reference inhibitors 7-DX (Figure 4b) and TPI [30]. It was found that, of the two isomers of compound **4**, the *R*-isomer interacted with the TP active site, and the oxygen atom from the carbonyl group in the C2 position of the hydantoin fragment created hydrogen bonds with two crucial TP amino acids: Ser186 and Arg171. Next, a large lipophilic fragment of the tetralin moiety of compound **4** was docked within a hydrophobic pocket of TP, comprising Leu117, Val177, Ile187, Phe210, and Leu220. The alkyl linker present in compound **4** created hydrophobic interactions with Tyr168. These interactions with the above-mentioned amino acids: Ser186, Arg171, as well as with Tyr168, were similar to the interactions of 7-DX with TP (Figure 4b). However, there was an absence of π–π stacking interactions with Tyr168 in the binding mode of compound **4**. Moreover, compound **4** exhibited a lack of interaction with Lys190, which is the third key residue for binding inhibitors to TP. The above deficiencies may explain the observed weak TP inhibitory activity of compound **4**. On the other hand, the *S*-isomer of compound **4** was not able to create any specific interactions with the most crucial amino acids. For the *S*-isomer, only a salt bridge between the protonated tertiary amine group from the piperazine ring and Asp164 was detected (Supplementary Figure S1).

Structure–activity relationship studies revealed that spirohydantoin plays a key role in antiproliferative activity. Although some of the 4-acetylphenylpiperazinylalkyl derivatives of spirohydantoin present modest cytotoxic activity, the investigation of *R* isomers of 3′,4′-dihydro-2′H-spiro[imidazolidine-4,1′-naphthalene]-2,5-dione have the potential to form the chemical base for the development of more potent compounds. This study reports on preliminary findings, as proof of the validity of the concept that the heterocyclic derivatives containing a 4-acetylphenylpiperazinylalkyl moiety are worthy of further investigation as promising pharmacophores for the design and development of compounds with cytotoxic activity against cancerous cells.

## 5. Conclusions

A new series of nitrogen-containing heterocyclic compounds, based on hydantoin and purine scaffolds, were designed and synthesized and their cytotoxic activity against selected cancer cell lines was evaluated. From the series of designed compounds tested, the anti-tumor activity of hydantoin derivatives with a 4-acetylphenylpiperazine-alkyl moiety stood out from the others in the series. Compound **4**, a derivative of 3′,4′-dihydro-2′H-spiro[imidazolidine-4,1′-naphthalene]-2,5-dione, was the most effective against SW480, SW620, and PC3 cancer cell lines. Moreover, compound **4** has high tumor-targeting selectivity. The overall results suggest that *R* isomers of 3′,4′-dihydro-2′H-spiro[imidazolidine-4,1′-naphthalene]-2,5-dione could be further modified to produce effective and selective anticancer agents.

## Figures and Tables

**Table 1:** Cytotoxic activity IC_50_ (µM) of compounds **1**–**14** was estimated by the MTT assay. Data are expressed as the mean ± SD.

Compd	Cell Lines						
	SW480 ^c^		SW 620 ^d^		PC3 ^e^		HaCaT ^f^
	IC_50_^a^	SI ^b^	IC_50_	SI	IC_50_	SI	IC_50_
**1**	54.18 ± 3.89	0.77	67.05 ± 5.24	0.62	57.62 ± 5.86	0.72	41.75 ± 7.62
**2**	40.68 ± 3.54	0.55	43.12 ± 4.41	0.52	36.23 ± 4.98	0.62	22.55 ± 2.69
**3**	129.35 ± 10.85	0.88	104.15 ± 7.36	1.09	64.45 ± 6.76	1.76	113.53 ± 8.93
**4**	16.8 ± 2.37	2.19	12.9 ± 3.16	2.85	20.58 ± 3.35	1.79	36.78 ± 5.32
**5**	102.09 ± 2.37	-	>200	-	>200	-	>200
**6**	53.67 ± 7.78	2.95	78.34 ± 4.76	2.02	95.12 ± 8.4	1.67	158.51 ± 14.12
**7**	103.33 ± 9.36	1.61	79.92 ± 5.12	2.08	88.9 ± 3.64	1.87	166.61 ± 1.58
**8**	62.17 ± 7.48	1.42	84.3 ± 4.87	1.05	42.8 ± 3.64	2.06	88.32 ± 3.27
**9**	153.34 ± 11.29	0.47	144.64 ± 3.76	0.50	101.26 ± 15.37	0.72	72.78 ± 6.08
**10**	>200	-	130.67 ± 12.64	0.73	86.12 ± 9.85	1.11	96.06 ± 5.32
**11**	>200	-	97.69 ± 10.74	0.60	60.28 ± 0.4	0.97	58.65 ± 4.91
**12**	>200	-	169.28 ± 22.91	0.49	71.85 ± 9.85	1.17	83.91 ± 4.20
**13**	>200	-	>200	-	94.2 ± 8.36	1.33	125.84 ± 14.35
**14**	>200	-	>200	-	90.97 ± 5.86	1.06	96.45 ± 4.93
**Doxorubicin**	0.29 ± 0.08	0.81	0.31 ± 0.07	0.76	0.59 ± 0.02	0.40	0.24 ± 0.03

^a^ IC_50_ (µM) concentration of the compound that corresponds to a 50% growth inhibition of the cell line (as compared to the control) after culturing the cells for 72 h with the individual compounds; ^b^ SI (Selectivity Index) = IC_50_ for the normal cell line/IC_50_ cancer cell line; ^c^ SW480—human primary colon cancer; ^d^ SW620—human metastatic colon cancer; ^e^ PC3—human metastatic prostate cancer cell line; ^f^ HaCaT—human immortal keratinocyte from adult human skin.

**Table 2:** Tumor-targeting selectivity of compound **4.** Data are expressed as the mean ± SD.

Compd	Cell Lines						
	SW480 ^c^		SW 620 ^dc^		PC3 ^e^		HMEC-1 ^f^
	IC_50_^a^	SI ^b^	IC_50_	SI	IC_50_	SI	IC_50_
**4**	16.8 ± 2.37	8.88	12.9 ± 3.16	11.57	20.58 ± 3.35	7.25	149.29 ± 10.63

^a^ IC_50_ (µM) concentration of the compound that corresponds to a 50% growth inhibition of cell line (as compared to the control) after cultured the cells for 72 h with the individual compound; ^b^ SI (Selectivity Index) = IC_50_ for normal cell line/IC_50_ cancer cell line; ^c^ SW480—human primary colon cancer; ^d^ SW620—human metastatic colon cancer; ^e^ PC3—human metastatic prostate cancer cell line; ^f^ HMEC-1—human immortal microvascular endothelial cells.

## Data Availability

Data Sharing is not applicable.

## Supplementary Materials

The following are available online at https://www.mdpi.com/article/10.3390/ma14154156/s1, ^1^H and ^13^C NMR spectra for compounds **1**–**14**, Figure S1: Binding mode of the *S*-isomer of compound **4** within TP active site.
