# Supplementary material for: Design, Synthesis, and In Vitro Antiproliferative Activity of Hydantoin and Purine Derivatives with the 4-Acetylphenylpiperazinylalkyl Moiety
Source: Materials (Basel). 2021 Jul 26;14(15):4156. doi: 10.3390/ma14154156 (PMC8347464; doi:10.3390/ma14154156)
Supplement: Supplementary file 1 [file materials-14-04156-s001.zip › materials-1282039-supplementary.pdf]

materials

Supplementary Materials

Design, Synthesis, and In Vitro Antiproliferative Activity of Hydantoin and Purine Derivatives with the 4-Acetylphenylpiperazinylalkyl Moiety

Agnieszka Zagórska [1,*], Anna Czopek [1], Anna Jaromin [2,*], Magdalena Mielczarek-Puta [3], Marta Struga [3], Dorota Stary [4] and Marek Bajda [4]

1 Department of Medicinal Chemistry, Faculty of Pharmacy, Jagiellonian University Medical College, Medyczna 9, 30-688 Kraków, Poland; anna.czopek@uj.edu.pl
2 Department of Lipids and Liposomes, Faculty of Biotechnology, University of Wroclaw, Joliot-Curie 14a, 50-383 Wroclaw, Poland
3 Chair and Department of Biochemistry, Medical University of Warsaw, Banacha 1, 02-097 Warsaw, Poland; magdalena.mielczarek-puta@wum.edu.pl (M.M.-P.); mstruga@wum.edu.pl (M.S.)
4 Department of Physicochemical Drug Analysis, Faculty of Pharmacy, Jagiellonian University Medical College, Medyczna 9, 30-688 Kraków, Poland; 1dorota.stary@student.uj.edu.pl (D.S.); marek.bajda@uj.edu.pl (M.B.)
* Correspondence: agnieszka.zagorska@uj.edu.pl (A.Z.); anna.jaromin@uwr.edu.pl (A.J.); Tel.: +48-2-62-05-450(A.Z.); Tel.: + 48-71-37-56-203 (A.J.)

Citation: Zagórska, A.; Czopek, A.; Jaromin, A.; Mielczarek-Puta, M.; Struga, M.; Stary, D.; Bajda, M. Design, Synthesis, and In Vitro Antiproliferative Activity of Hydantoin and Purine Derivatives with the 4-Acetylphenylpiperazinylalkyl Moiety. *Materials* **2021**, *14*, 4156. https://doi.org/10.3390/ma14154156

Academic Editor: Abdelwahab Omri

Received: 16 June 2021
Accepted: 23 July 2021
Published: 26 July 2021

Publisher's Note: MDPI stays neutral with regard to jurisdictional claims in published maps and institutional affiliations.

Copyright: © 2021 by the authors. Licensee MDPI, Basel, Switzerland. This article is an open access article distributed under the terms and conditions of the Creative Commons Attribution (CC BY) license (http://creativecommons.org/licenses/by/4.0/).

Herein, we presented proton and carbon nuclear magnetic resonance (^{1}H and ^{13}C NMR) spectra for compounds **1–14**, which were recorded on a Varian Mercury spectrometer (Varian Inc., Palo Alto, USA) or on an FT-NMR 500 MHz spectrometer (Joel Ltd.) as well as Figure S1 with binding mode of the *S*-isomer of compound **4**.

Compound **1**

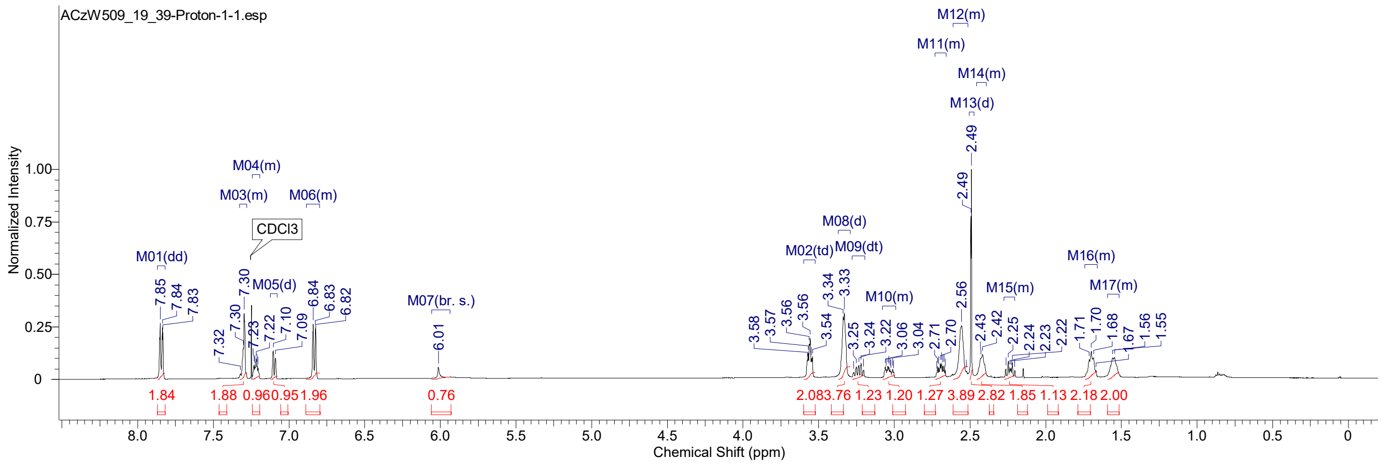
ACzW509_19_39-Proton-1-1.esp
Normalized Intensity
CDCl3
Chemical Shift (ppm)

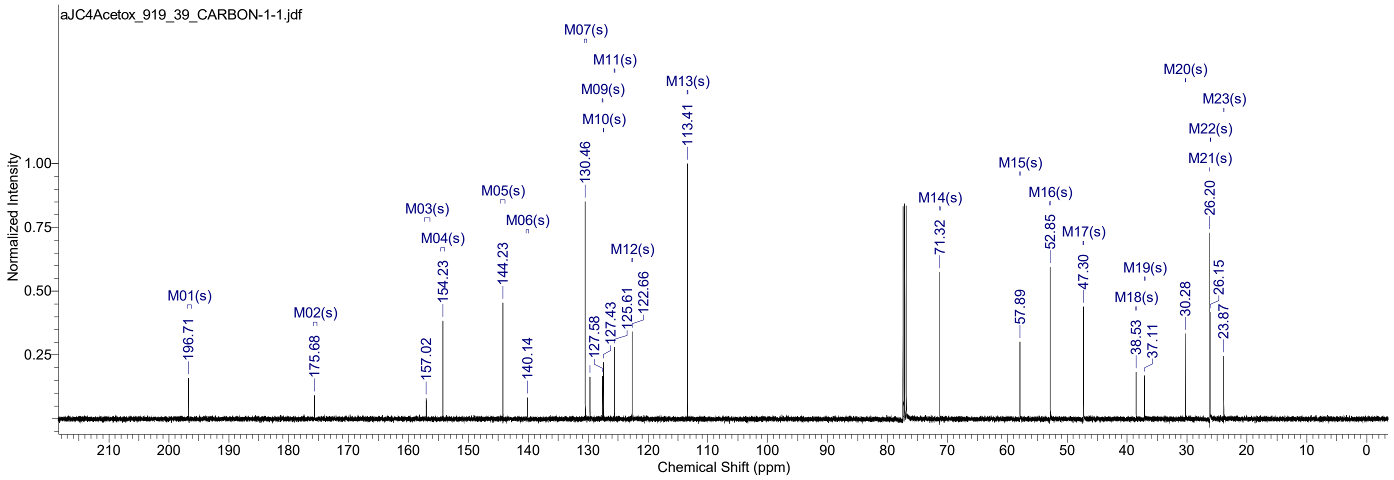
aJC4Acetox_919_39_CARBON-1-1.jdf
Normalized Intensity
Chemical Shift (ppm)

Compound **2**

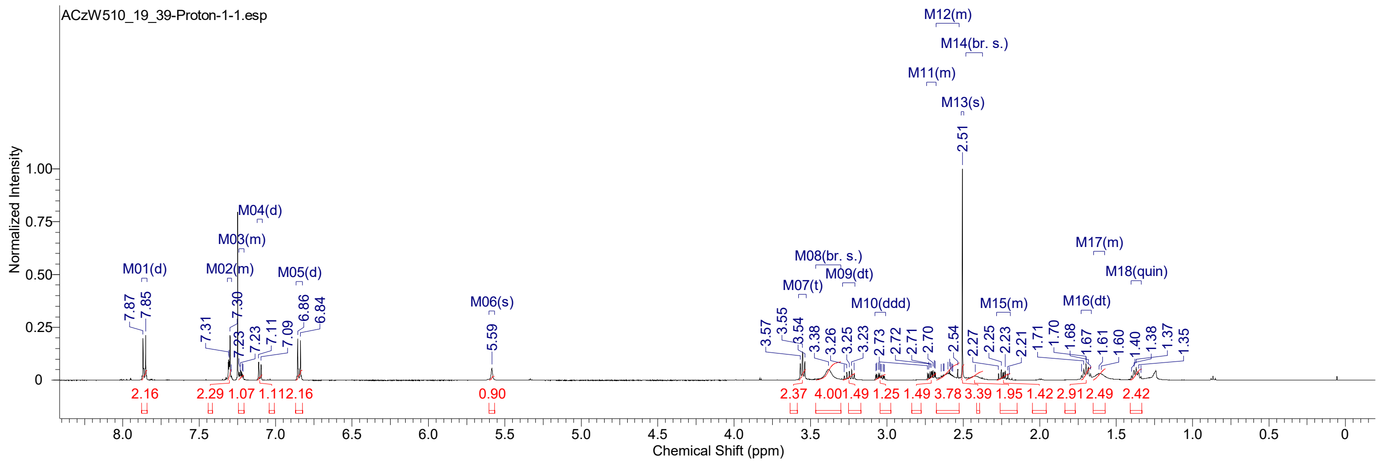
ACzW510_19_39-Proton-1-1.esp
Normalized Intensity
Chemical Shift (ppm)
M01(d)
M02(m)
M03(m)
M04(d)
M05(d)
M06(s)
M07(t)
M08(br. s.)
M09(dt)
M10(ddd)
M11(m)
M12(m)
M13(s)
M14(br. s.)
M15(m)
M16(dt)
M17(m)
M18(quin)
2.16 2.29 1.07 1.11 2.16 0.90 2.37 4.00 1.49 1.25 1.49 3.78 3.39 1.95 1.42 2.91 2.49 2.42

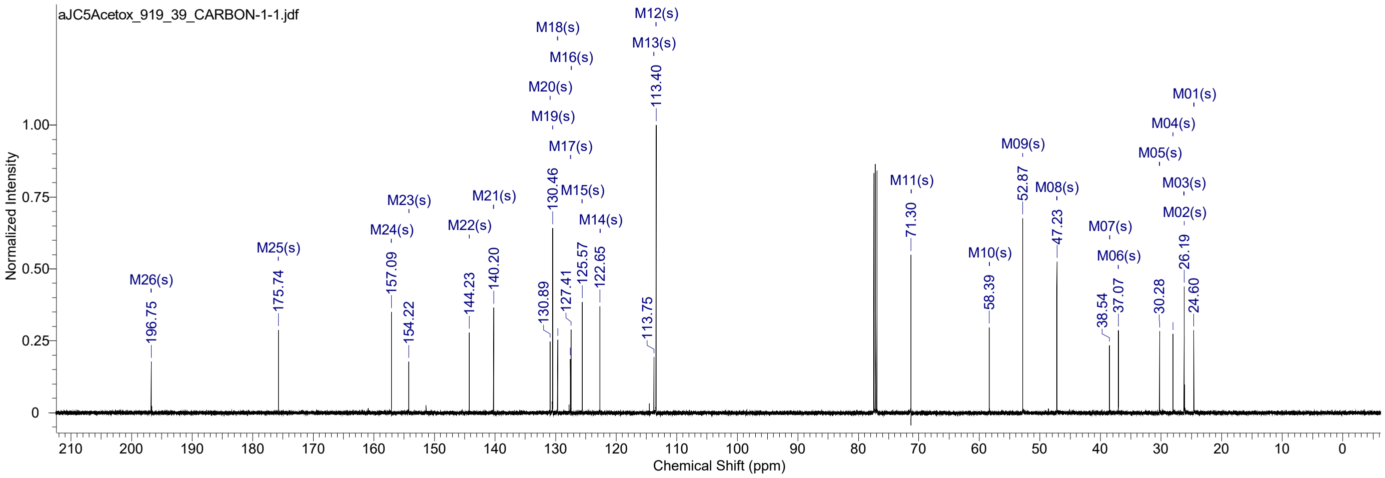
aJC5Acetox_919_39_CARBON-1-1.jdf
Normalized Intensity
Chemical Shift (ppm)
M26(s) 196.75
M25(s) 175.74
M24(s) 157.09
M23(s) 154.22
M22(s) 144.23
M21(s) 140.20
130.89
130.46
127.41
125.57
122.65
113.75
113.40
M11(s) 71.30
M10(s) 58.39
M09(s) 52.87
M08(s) 47.23
M07(s) 38.54
M06(s) 37.07
30.28
26.19
24.60

Compound **3**

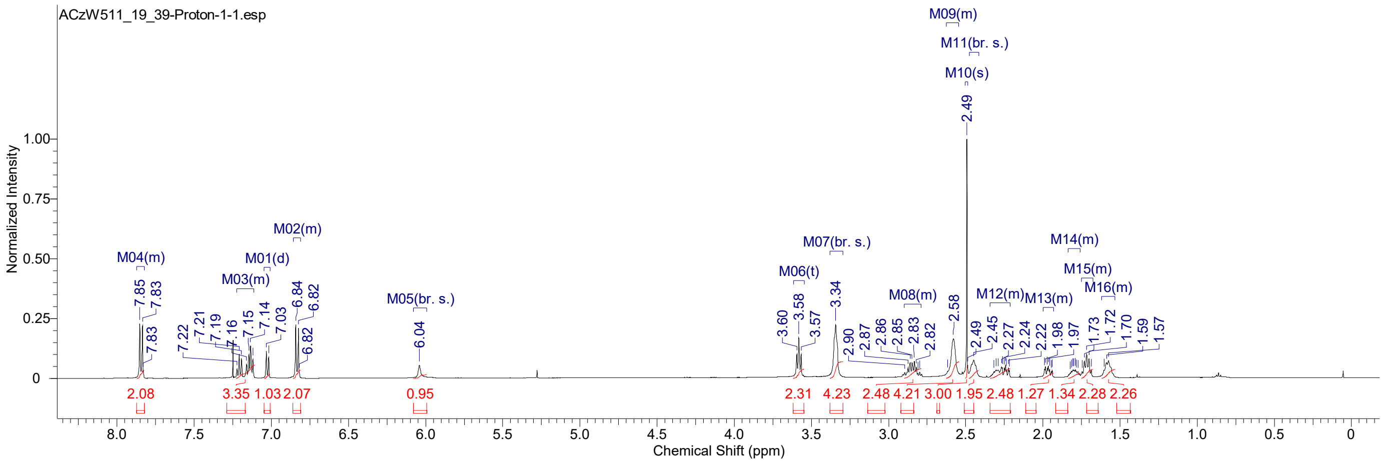
ACzW511_19_39-Proton-1-1.esp
Normalized Intensity
Chemical Shift (ppm)
M01(d)
M02(m)
M03(m)
M04(m)
M05(br. s.)
M06(t)
M07(br. s.)
M08(m)
M09(m)
M10(s)
M11(br. s.)
M12(m)
M13(m)
M14(m)
M15(m)
M16(m)
2.08 3.35 1.03 2.07 0.95 2.31 4.23 2.48 4.21 3.00 1.95 2.48 1.27 1.34 2.28 2.26

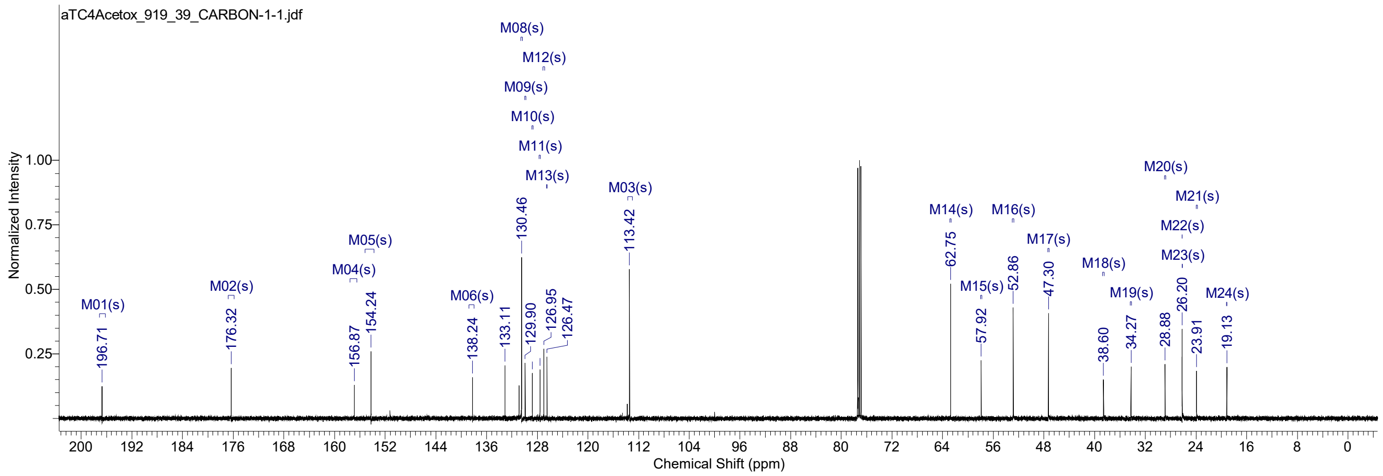
aTC4Acetox_919_39_CARBON-1-1.jdf
Normalized Intensity
Chemical Shift (ppm)
196.71
176.32
156.87
154.24
138.24
133.11
130.46
129.90
126.95
126.47
113.42
62.75
57.92
52.86
47.30
38.60
34.27
28.88
26.20
23.91
19.13

Compound **4**

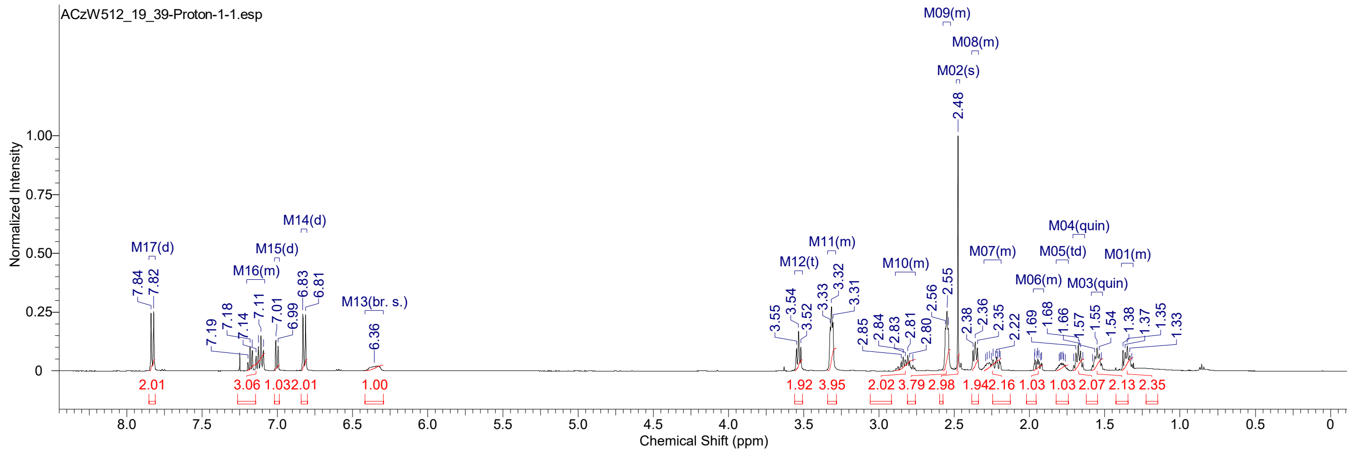
ACzW512_19_39-Proton-1-1.esp
Normalized Intensity
Chemical Shift (ppm)
M17(d)
M16(m)
M15(d)
M14(d)
M13(br. s.)
M12(t)
M11(m)
M10(m)
M09(m)
M08(m)
M02(s)
M07(m)
M06(m)
M05(td)
M04(quin)
M03(quin)
M01(m)
2.01 3.06 1.03 2.01 1.00 1.92 3.95 2.02 3.79 2.98 1.94 2.16 1.03 1.03 2.07 2.13 2.35

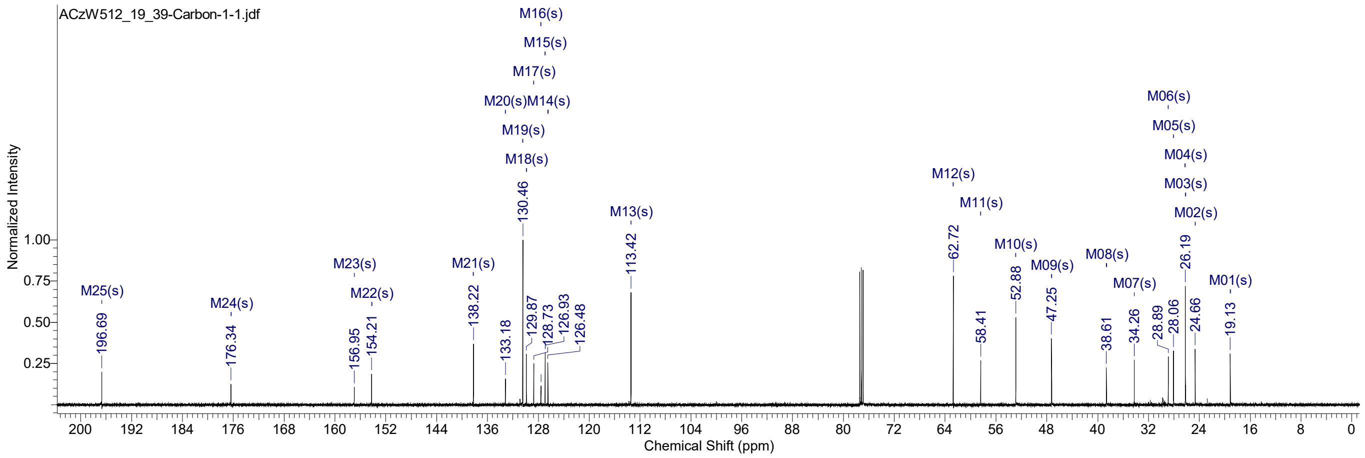
ACzW512_19_39-Carbon-1-1.jdf
Normalized Intensity
Chemical Shift (ppm)
M25(s) 196.69
M24(s) 176.34
M23(s) 156.95
M22(s) 154.21
M21(s) 138.22
M16(s)
M15(s)
M17(s)
M20(s)M14(s)
M19(s)
M18(s)
130.46 133.18 129.87 128.73 126.93 126.48
M13(s) 113.42
M12(s) 62.72
M11(s) 58.41
M10(s) 52.88
M09(s) 47.25
M08(s) 38.61
M07(s) 34.26
M06(s)
M05(s)
M04(s)
M03(s)
M02(s)
28.89 28.06 26.19 24.66
M01(s) 19.13

Compound **5**

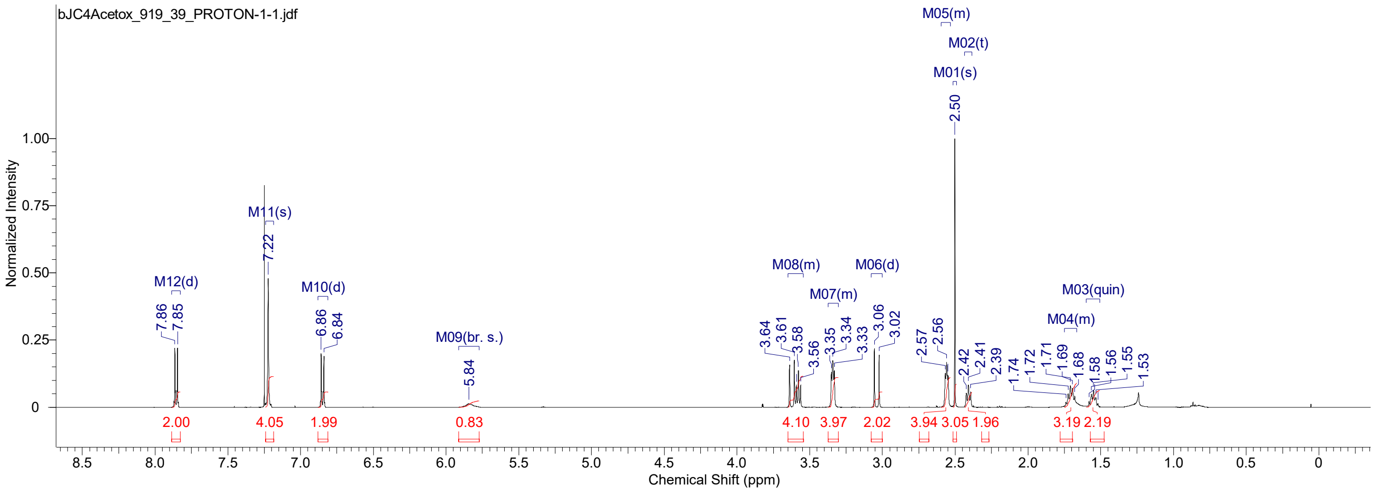
bJC4Acetox_919_39_PROTON-1-1.jdf
Normalized Intensity
Chemical Shift (ppm)

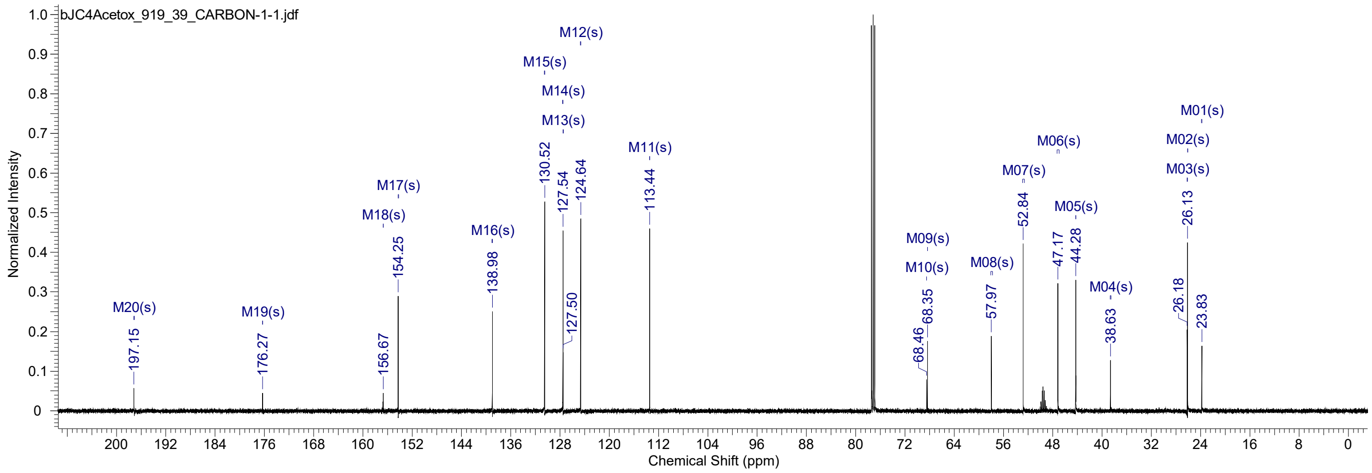
bJC4Acetox_919_39_CARBON-1-1.jdf
Normalized Intensity
Chemical Shift (ppm)

Compound **6**

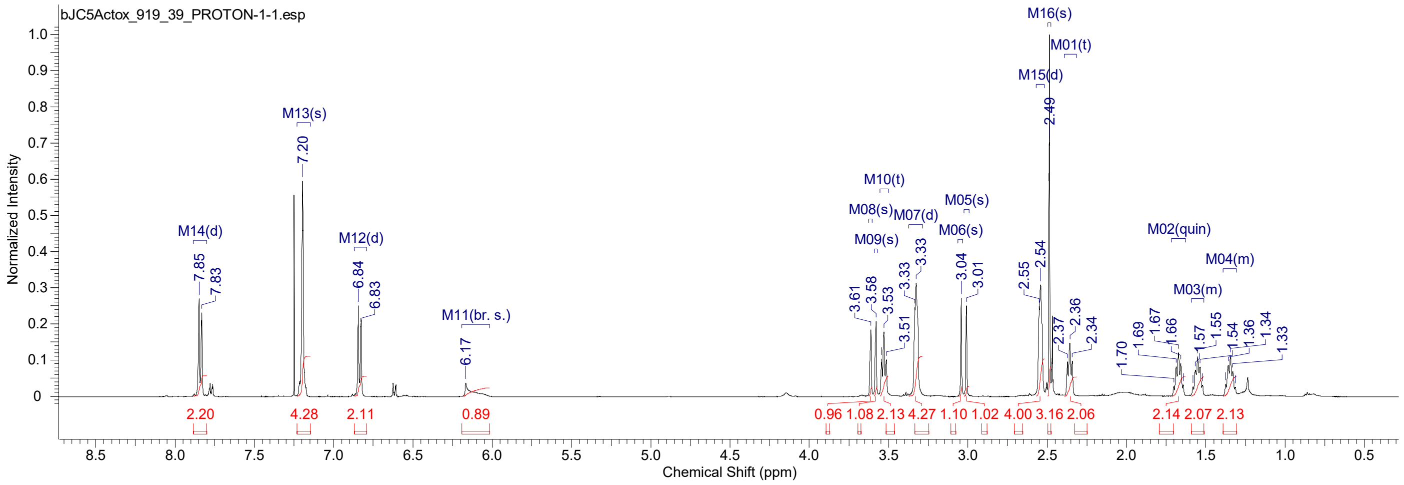
bJC5Actox_919_39_PROTON-1-1.esp
Normalized Intensity
Chemical Shift (ppm)
M16(s)
M01(t)
M15(d)
M13(s)
M14(d)
M12(d)
M11(br. s.)
M10(t)
M08(s)
M07(d)
M09(s)
M05(s)
M06(s)
M02(quin)
M04(m)
M03(m)
7.85 7.83 7.20 6.84 6.83 6.17 3.61 3.58 3.53 3.51 3.33 3.33 3.04 3.01 2.55 2.54 2.49 2.37 2.36 2.34 1.70 1.69 1.67 1.66 1.57 1.55 1.54 1.36 1.34 1.33
2.20 4.28 2.11 0.89 0.96 1.08 2.13 4.27 1.10 1.02 4.00 3.16 2.06 2.14 2.07 2.13

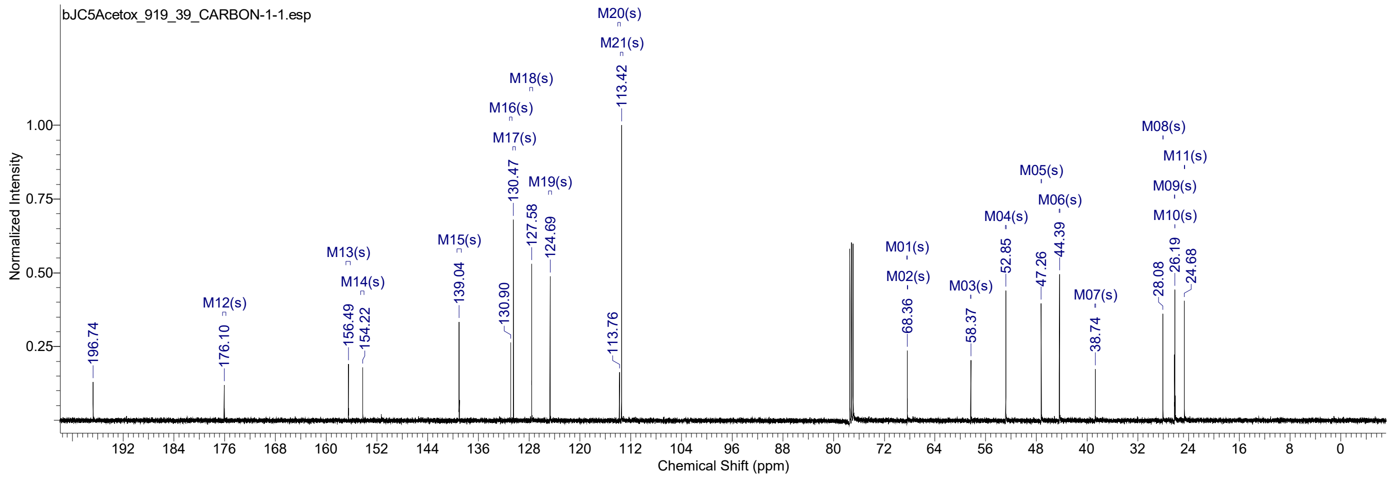
bJC5Acetox_919_39_CARBON-1-1.esp
Normalized Intensity
Chemical Shift (ppm)
M20(s)
M21(s)
M18(s)
M16(s)
M17(s)
M19(s)
M15(s)
M13(s)
M14(s)
M12(s)
M01(s)
M02(s)
M03(s)
M04(s)
M05(s)
M06(s)
M07(s)
M08(s)
M11(s)
M09(s)
M10(s)
196.74 176.10 156.49 154.22 139.04 130.90 130.47 127.58 124.69 113.76 113.42 68.36 58.37 52.85 47.26 44.39 38.74 28.08 26.19 24.68

Compound **7**

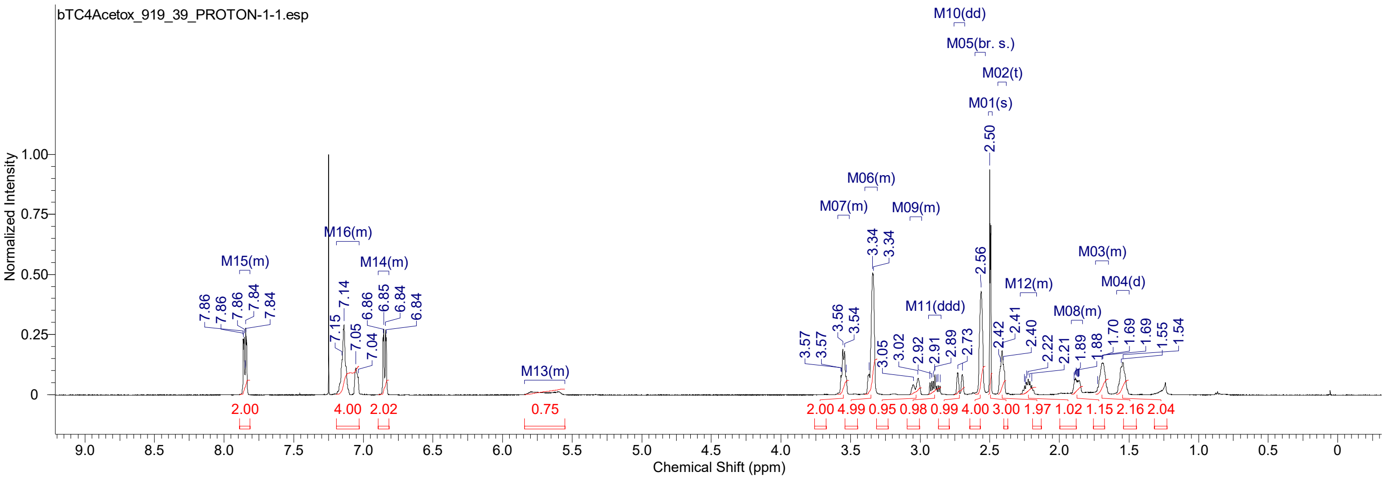
bTC4Acetox_919_39_PROTON-1-1.esp
Normalized Intensity
Chemical Shift (ppm)
M15(m) 7.86 7.86 7.86 7.84 7.84
M16(m) 7.15 7.14 7.05 7.04
M14(m) 6.86 6.85 6.84 6.84
M13(m)
M07(m) 3.57 3.57 3.56 3.54
M06(m) 3.34 3.34
M09(m) 3.05 3.02 2.92
M11(ddd) 2.91 2.89 2.73
M10(dd)
M05(br. s.) 2.56
M02(t)
M01(s) 2.50
M12(m) 2.42 2.41 2.40 2.22 2.21
M08(m) 1.89 1.88
M03(m) 1.70 1.69 1.69
M04(d) 1.55 1.54
2.00 4.00 2.02 0.75 2.00 4.99 0.95 0.98 0.99 4.00 3.00 1.97 1.02 1.15 2.16 2.04

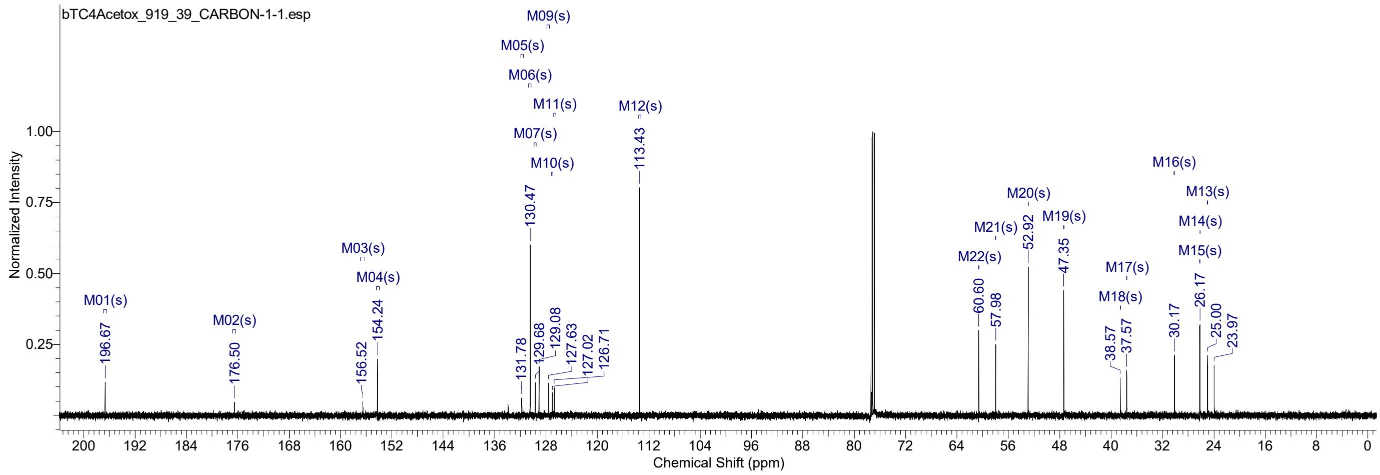
bTC4Acetox_919_39_CARBON-1-1.esp
Normalized Intensity
Chemical Shift (ppm)
M01(s) 196.67
M02(s) 176.50
M03(s) 156.52
M04(s) 154.24
M09(s) M05(s) M06(s) M11(s) M07(s) M10(s)
131.78 130.47 129.68 129.08 127.63 127.02 126.71
M12(s) 113.43
M22(s) 60.60
M21(s) 57.98
M20(s) 52.92
M19(s) 47.35
M17(s) 38.57
M18(s) 37.57
M16(s) 30.17
M13(s) 26.17
M14(s) 25.00
M15(s) 23.97

Compound **8**

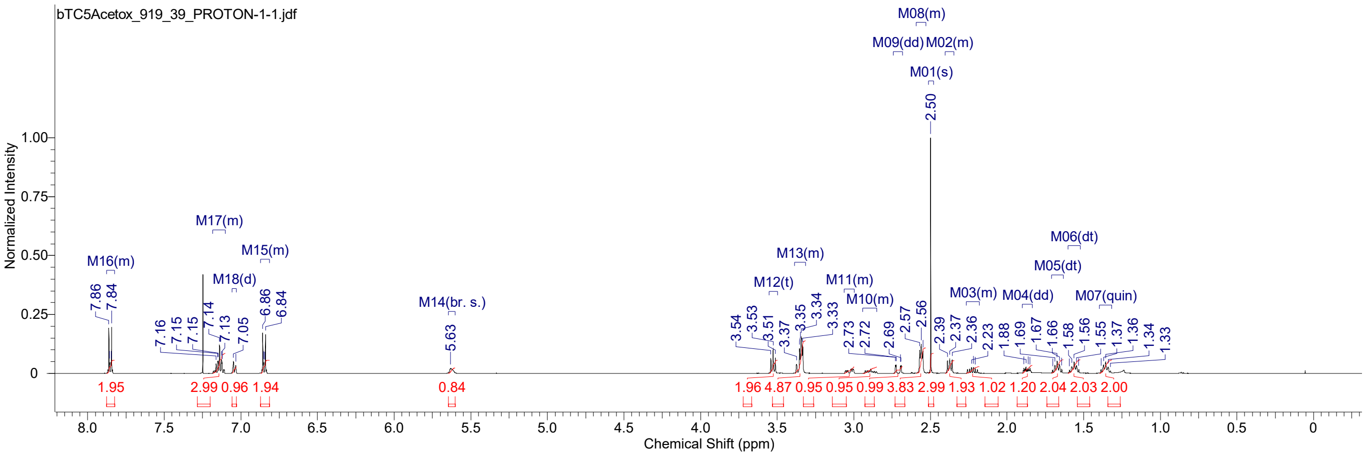
bTC5Acetox_919_39_PROTON-1-1.jdf
Normalized Intensity
Chemical Shift (ppm)
M08(m)
M09(dd) M02(m)
M01(s)
M16(m)
M17(m)
M18(d)
M15(m)
M14(br. s.)
M12(t)
M13(m)
M11(m)
M10(m)
M03(m) M04(dd)
M05(dt)
M06(dt)
M07(quin)
7.86 7.84 7.16 7.15 7.15 7.14 7.13 7.05 6.86 6.84 5.63 3.54 3.53 3.51 3.37 3.35 3.34 3.33 2.73 2.72 2.69 2.57 2.56 2.50 2.39 2.37 2.36 2.23 1.88 1.69 1.67 1.66 1.58 1.56 1.55 1.37 1.36 1.34 1.33
1.95 2.99 0.96 1.94 0.84 1.96 4.87 0.95 0.95 0.99 3.83 2.99 1.93 1.02 1.20 2.04 2.03 2.00

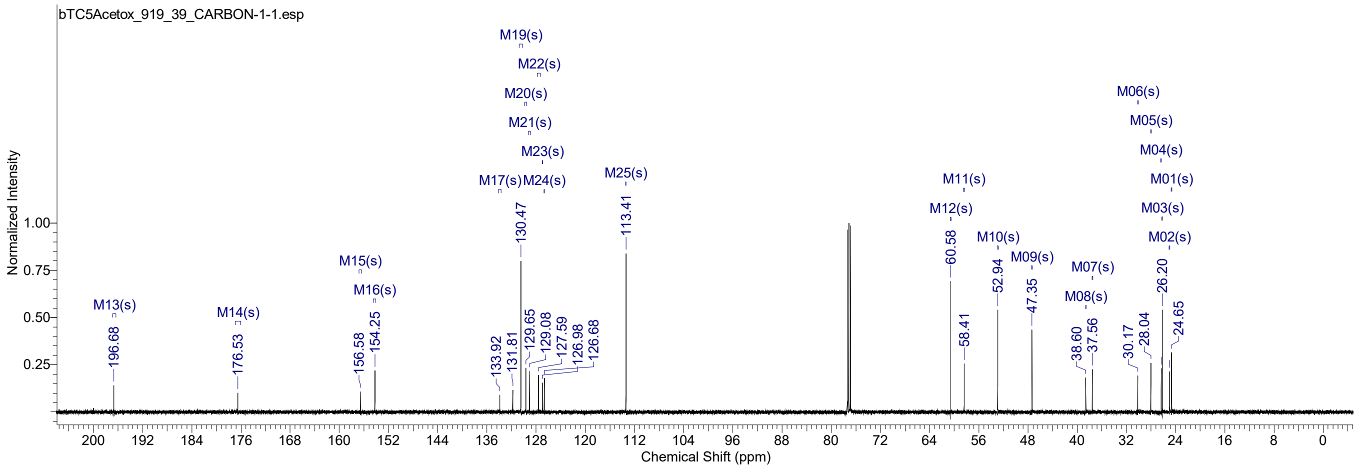
bTC5Acetox_919_39_CARBON-1-1.esp
Normalized Intensity
Chemical Shift (ppm)
M19(s)
M22(s)
M20(s)
M21(s)
M23(s)
M17(s) M24(s)
M25(s)
M13(s)
M14(s)
M15(s)
M16(s)
M11(s)
M12(s)
M10(s)
M09(s)
M07(s)
M08(s)
M06(s)
M05(s)
M04(s)
M01(s)
M03(s)
M02(s)
196.68 176.53 156.58 154.25 133.92 131.81 130.47 129.65 129.08 127.59 126.98 126.68 113.41 60.58 58.41 52.94 47.35 38.60 37.56 30.17 28.04 26.20 24.65

Compound **9**

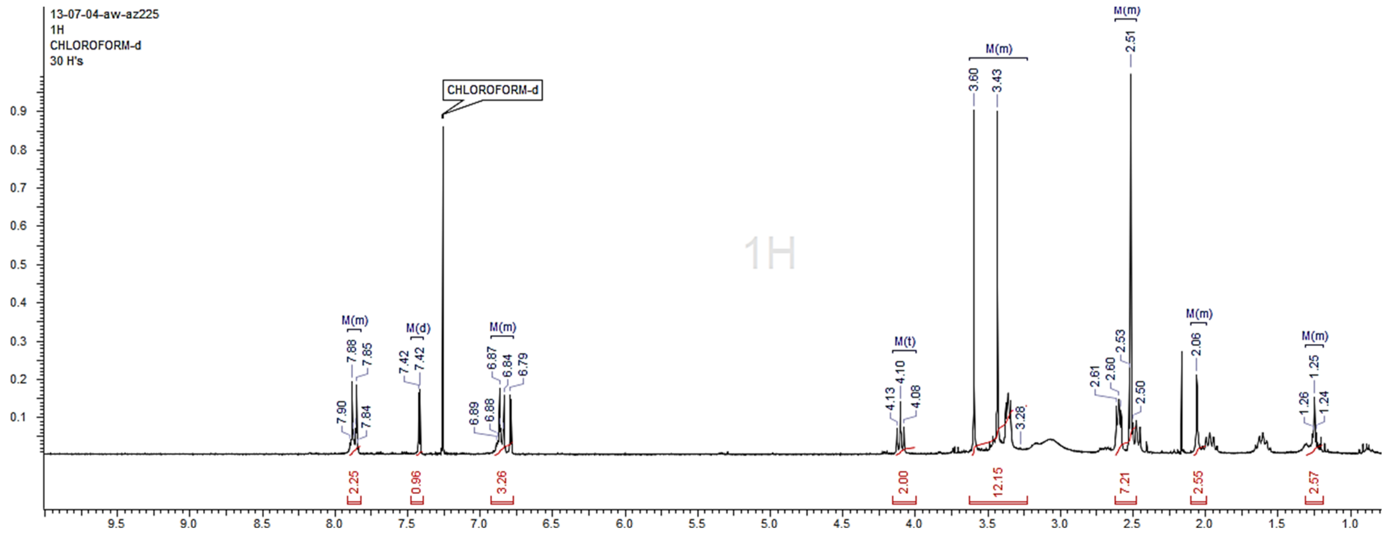
13-07-04-aw-az225
1H
CHLOROFORM-d
30 H's
CHLOROFORM-d
1H
M(m)
M(d)
M(t)
2.25
0.96
3.26
2.00
12.15
7.21
2.55
2.57

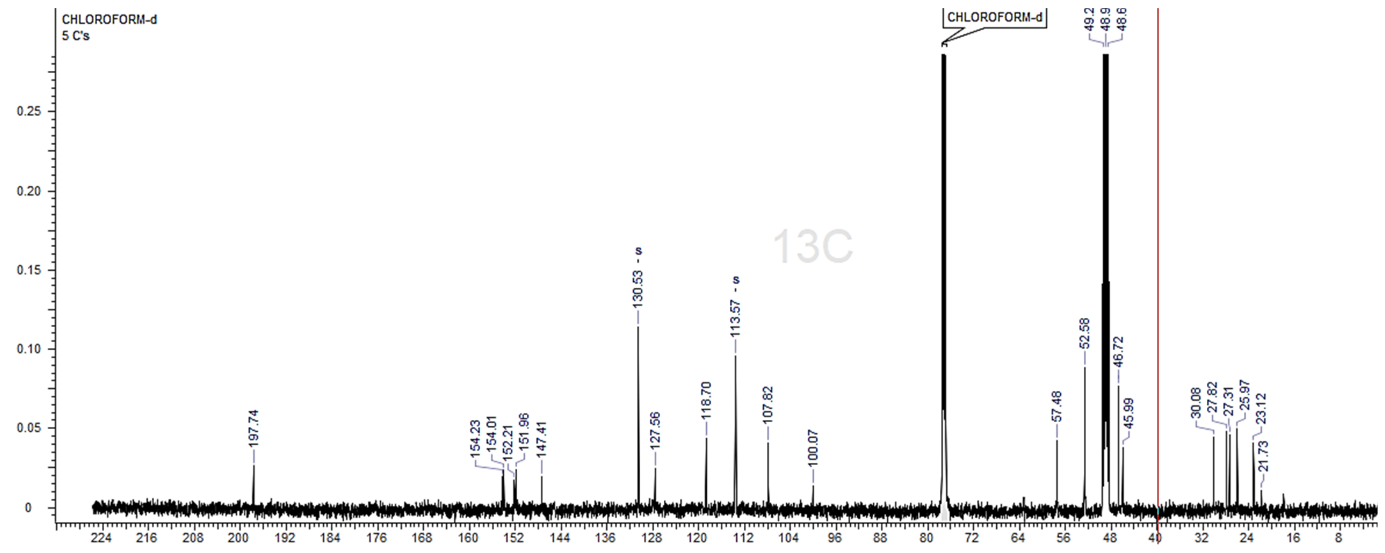
CHLOROFORM-d
5 C's
CHLOROFORM-d
13C
197.74
154.23
154.01
152.21
151.96
147.41
130.53
127.56
118.70
113.57
107.82
100.07
57.48
52.58
46.72
45.99
30.08
27.82
27.31
25.97
23.12
21.73

Compound **10**

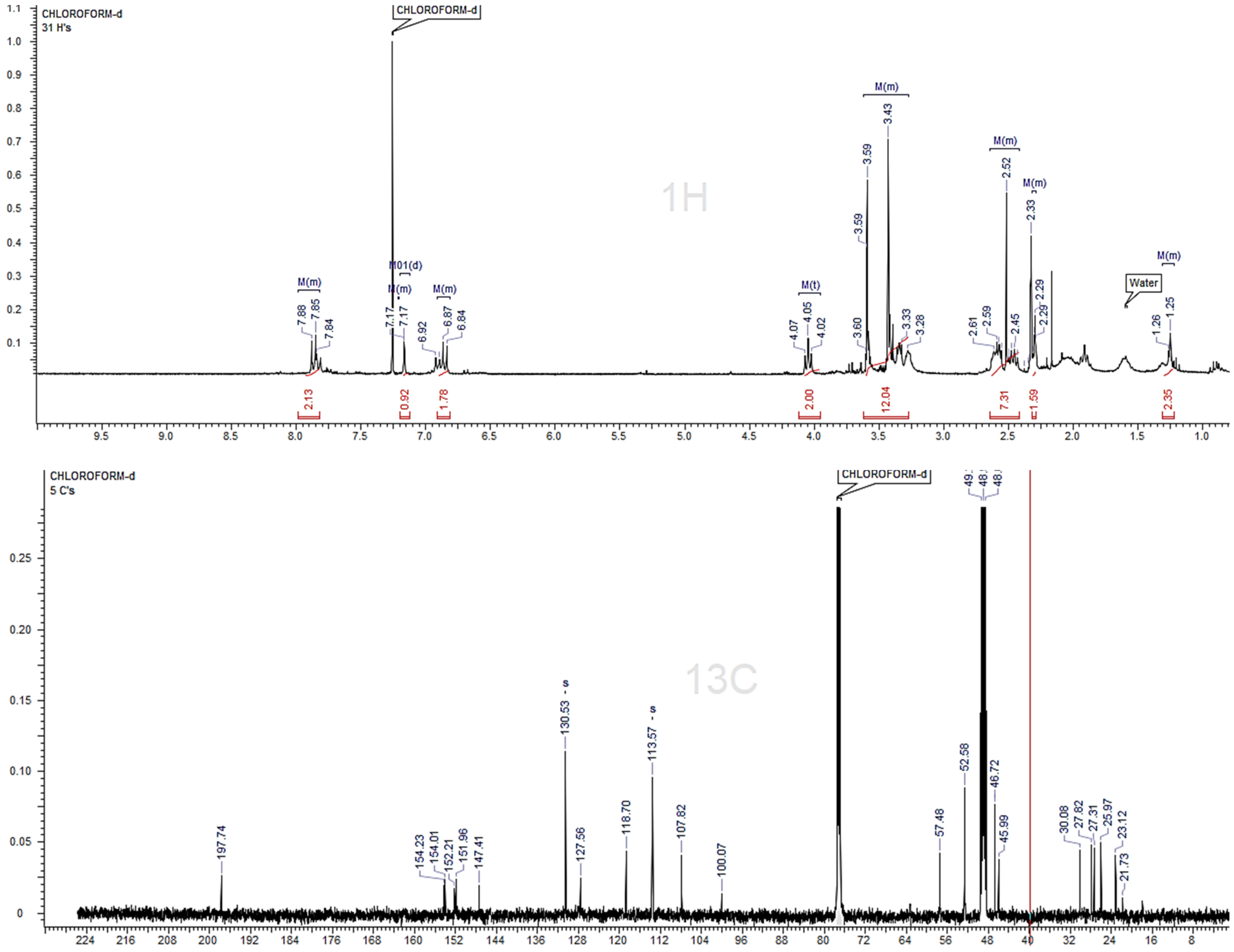
CHLOROFORM-d
31 H's
CHLOROFORM-d
1H
M(m)
7.88
7.85
7.84
M01(d)
M(m)
7.17
7.17
M(m)
6.92
6.87
6.84
M(t)
4.07
4.05
4.02
M(m)
3.59
3.59
3.43
3.60
3.33
3.28
M(m)
2.52
2.61
2.59
2.45
M(m)
2.33
2.29
2.29
Water
M(m)
1.26
1.25
2.13
0.92
1.78
2.00
12.04
7.31
1.59
2.35
CHLOROFORM-d
5 C's
CHLOROFORM-d
13C
197.74
154.23
154.01
152.21
151.96
147.41
130.53 - s
127.56
118.70
113.57 - s
107.82
100.07
57.48
52.58
46.72
45.99
30.08
27.82
27.31
25.97
23.12
21.73

Compound **11**

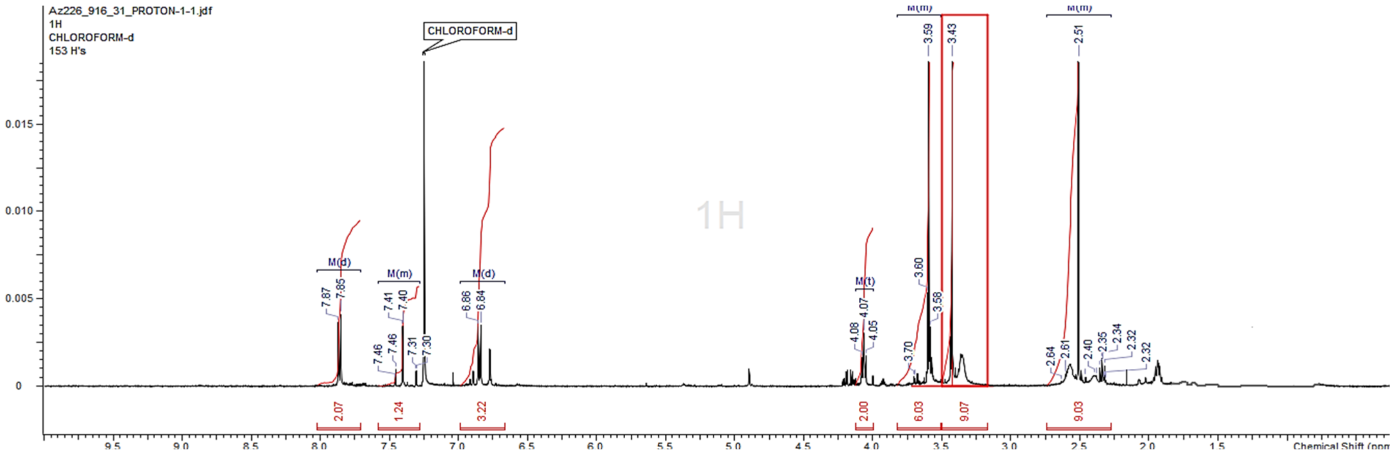
Az226_916_31_PROTON-1-1.jdf
1H
CHLOROFORM-d
153 H's
CHLOROFORM-d
1H
M(d)
M(m)
M(d)
M(t)
M(m)
M(m)
7.87
7.85
7.46
7.46
7.41
7.40
7.31
7.30
6.86
6.84
4.08
4.07
4.05
3.70
3.60
3.59
3.58
3.43
2.64
2.61
2.51
2.40
2.35
2.34
2.32
2.32
2.07
1.24
3.22
2.00
6.03
9.07
9.03
Chemical Shift (ppm)

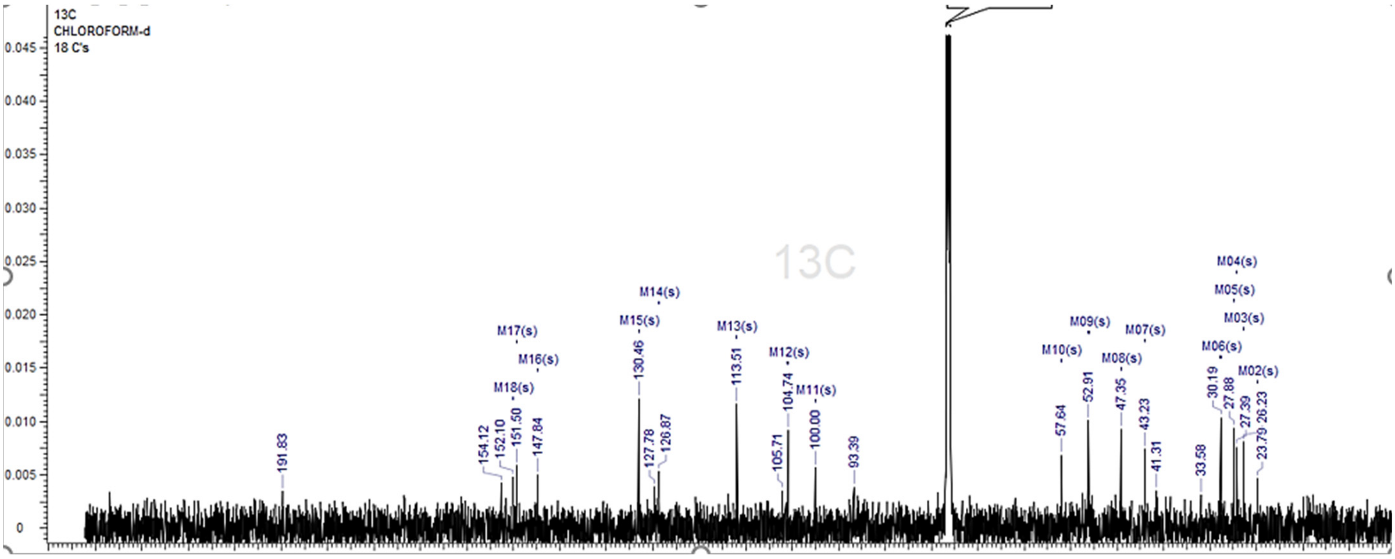
13C
CHLOROFORM-d
18 C's
13C
191.83
154.12
152.10
151.50
147.84
130.46
127.78
126.87
113.51
105.71
104.74
100.00
93.39
57.64
52.91
47.35
43.23
41.31
33.58
30.19
27.88
27.39
26.23
23.79
M18(s)
M17(s)
M16(s)
M15(s)
M14(s)
M13(s)
M12(s)
M11(s)
M10(s)
M09(s)
M08(s)
M07(s)
M06(s)
M05(s)
M04(s)
M03(s)
M02(s)

Compound **12**

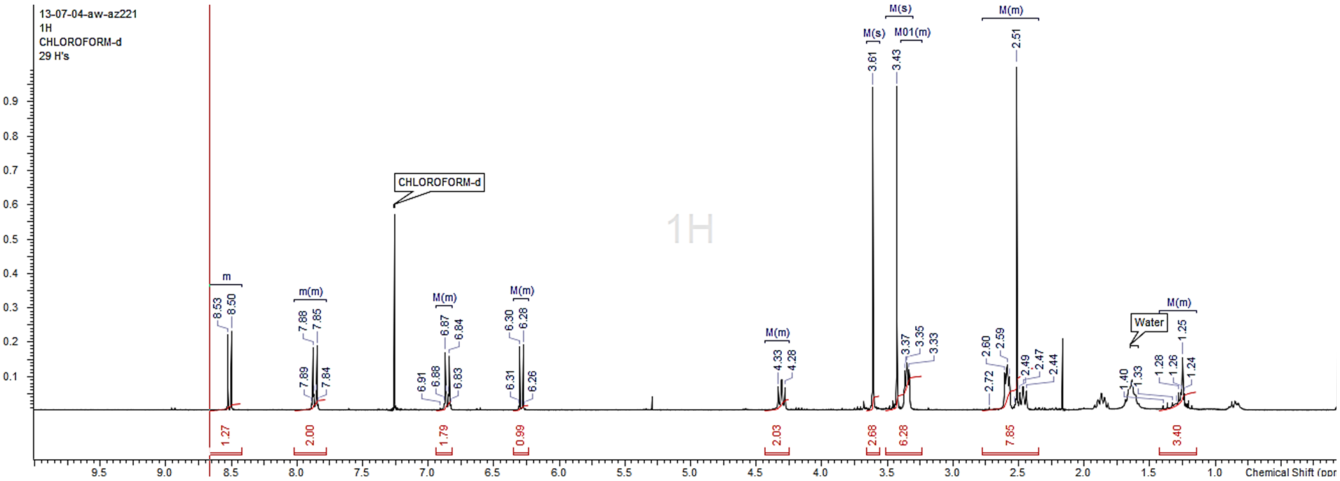
13-07-04-aw-az221
1H
CHLOROFORM-d
29 H's
CHLOROFORM-d
1H
Water
8.53
8.50
7.89
7.88
7.85
7.84
6.91
6.88
6.87
6.84
6.83
6.31
6.30
6.28
6.26
4.33
4.28
3.61
3.43
3.37
3.35
3.33
2.72
2.60
2.59
2.51
2.49
2.47
2.44
1.40
1.33
1.28
1.26
1.25
1.24
1.27
2.00
1.79
0.99
2.03
2.68
6.28
7.85
3.40
Chemical Shift (ppr

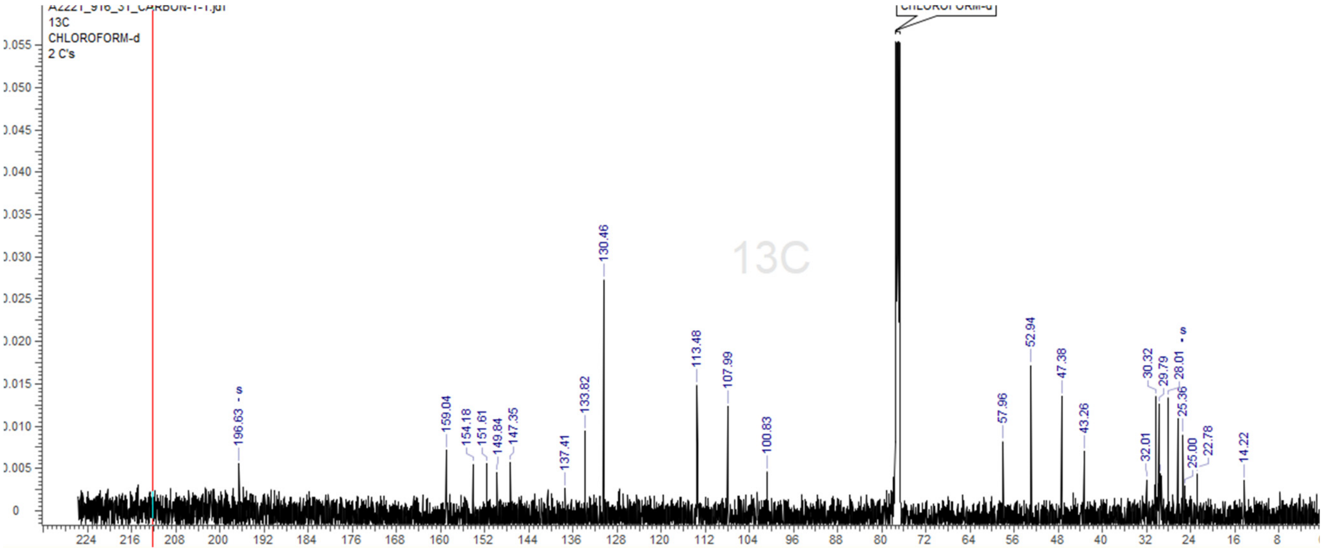
13C
CHLOROFORM-d
2 C's
13C
196.63
159.04
154.18
151.61
149.84
147.35
137.41
133.82
130.46
113.48
107.99
100.83
57.96
52.94
47.38
43.26
32.01
30.32
29.79
28.01
25.36
25.00
22.78
14.22

Compound **13**

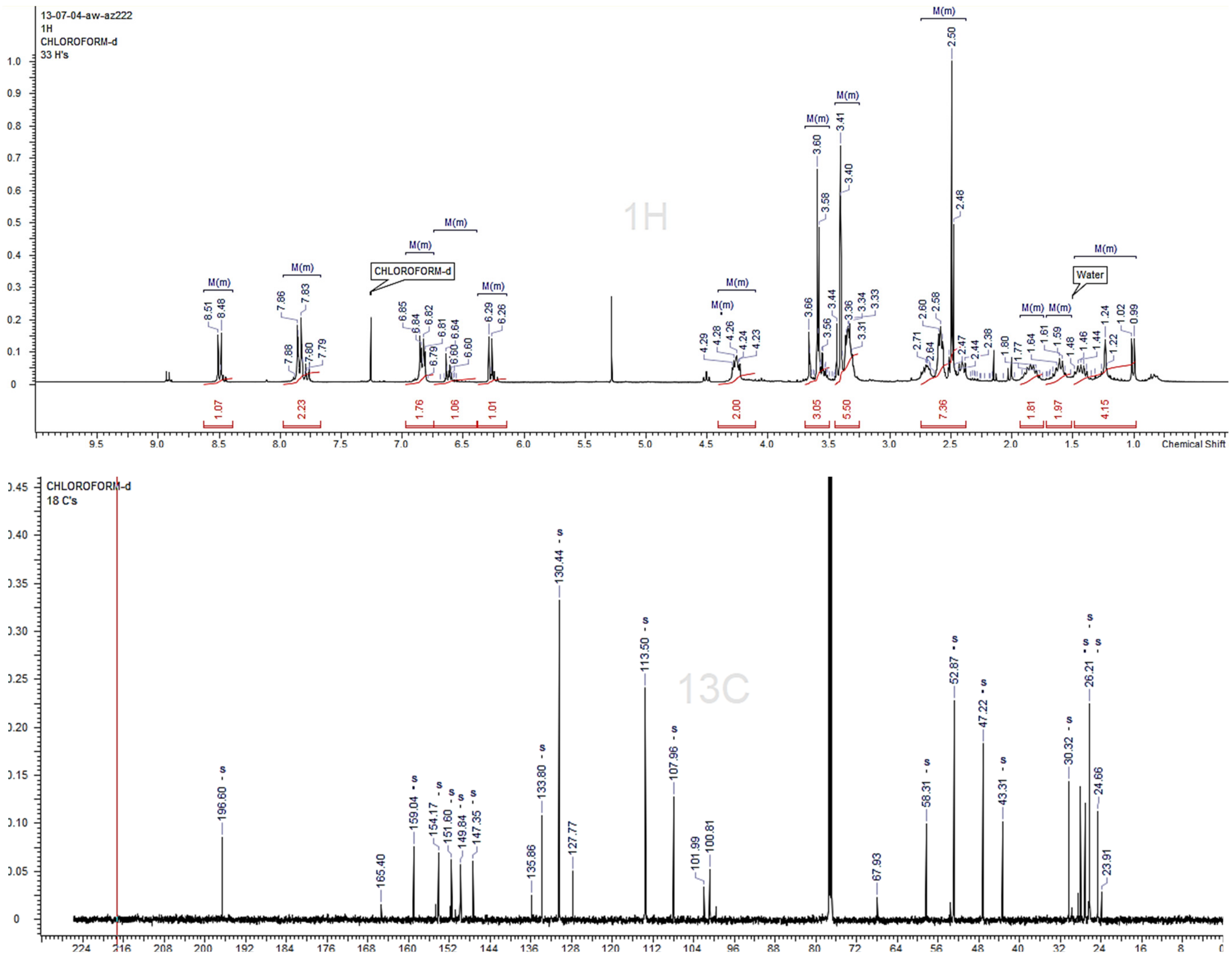
13-07-04-aw-az222
1H
CHLOROFORM-d
33 H's
1H
CHLOROFORM-d
Water
M(m)
8.51 8.48
7.88 7.86 7.83 7.80 7.79
6.85 6.84 6.82 6.81 6.79 6.60 6.64 6.60
6.29 6.26
4.29 4.28 4.26 4.24 4.23
3.66 3.60 3.58 3.56 3.44 3.41 3.40 3.36 3.34 3.33 3.31
2.71 2.64 2.60 2.58 2.50 2.48 2.47 2.44 2.38
1.80 1.77 1.64 1.61 1.59 1.48 1.46 1.44 1.24 1.22 1.02 0.99
1.07 2.23 1.76 1.06 1.01 2.00 3.05 5.50 7.36 1.81 1.97 4.15
Chemical Shift
CHLOROFORM-d
18 C's
13C
196.60 165.40 159.04 154.17 151.60 149.84 147.35 135.86 133.80 130.44 127.77 113.50 107.96 101.99 100.81 67.93 58.31 52.87 47.22 43.31 30.32 26.21 24.66 23.91
s

Compound **14**

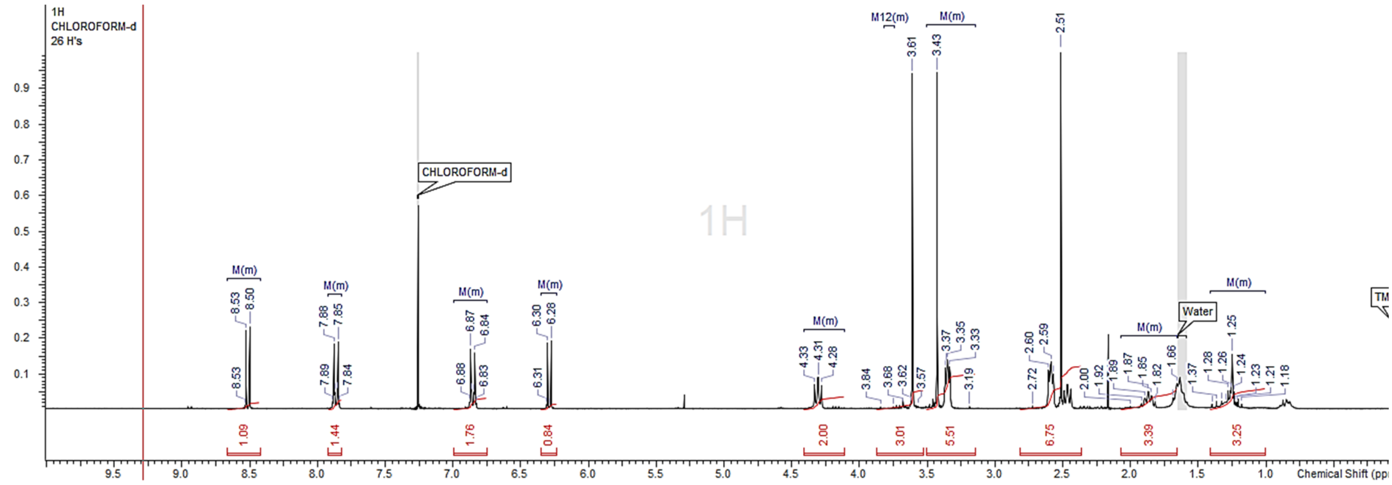
1H
CHLOROFORM-d
26 H's
CHLOROFORM-d
1H
Water
M(m)
M12(m)
8.53
8.50
7.89
7.88
7.85
7.84
6.88
6.87
6.84
6.83
6.31
6.30
6.28
4.33
4.31
4.28
3.84
3.68
3.62
3.61
3.57
3.43
3.37
3.35
3.33
3.19
2.72
2.60
2.59
2.51
2.00
1.92
1.89
1.87
1.85
1.82
1.66
1.37
1.28
1.26
1.25
1.24
1.23
1.21
1.18
1.09
1.44
1.76
0.84
2.00
3.01
5.51
6.75
3.39
3.25
Chemical Shift (pp

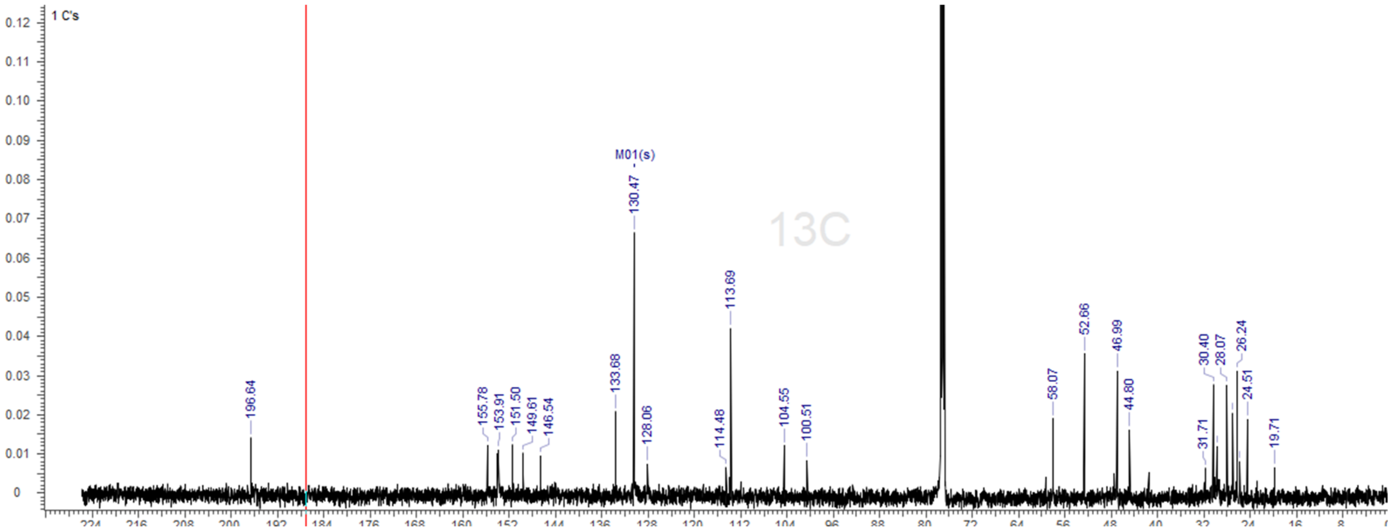
1 C's
13C
M01(s)
196.64
155.78
153.91
151.50
149.61
146.54
133.68
130.47
128.06
114.48
113.69
104.55
100.51
58.07
52.66
46.99
44.80
31.71
30.40
28.07
26.24
24.51
19.71

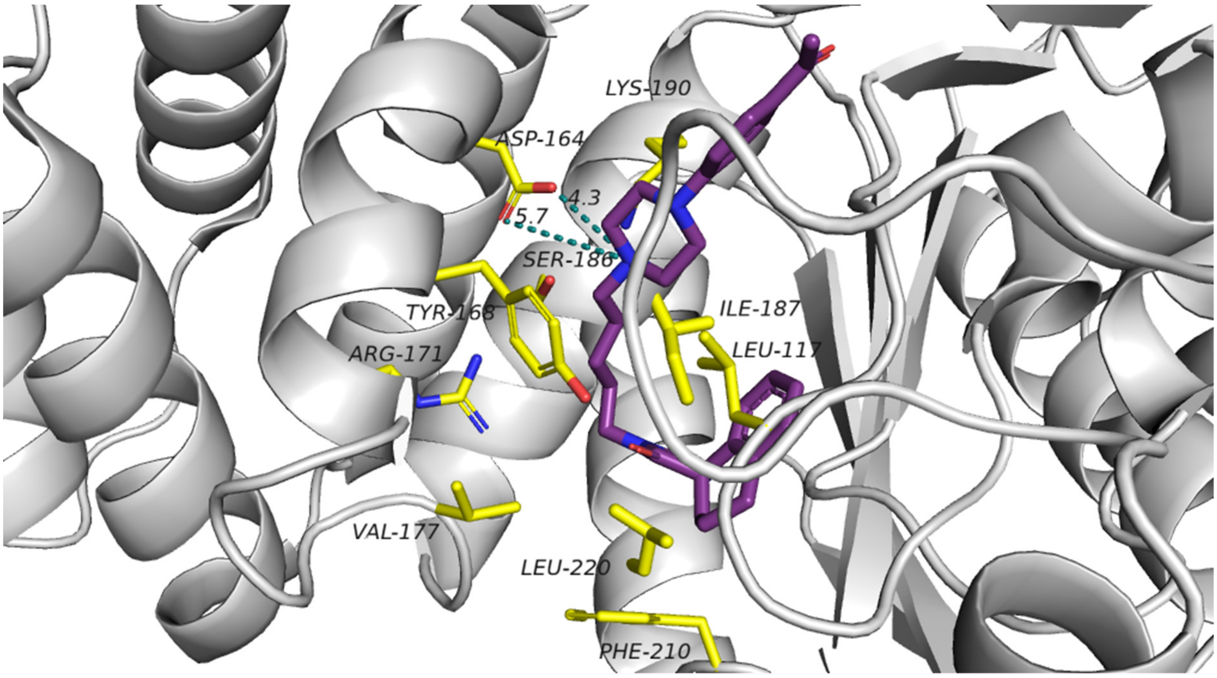

Figure S1. Binding mode of the *S*-isomer of compound 4 within TP active site. Residues colored in yellow. Salt bridges are presented as blue dashes.
